# Functional and Compositional Changes in *Sirex noctilio* Gut Microbiome in Different Habitats: Unraveling the Complexity of Invasive Adaptation

**DOI:** 10.3390/ijms25052526

**Published:** 2024-02-21

**Authors:** Jiale Li, Ningning Fu, Ming Wang, Chenglong Gao, Bingtao Gao, Lili Ren, Jing Tao, Youqing Luo

**Affiliations:** 1 Beijing Key Laboratory for Forest Pest Control, Beijing Forestry University, Beijing 100083, China; ljl670509@163.com (J.L.); funingning2012@sina.com (N.F.); 13020028768@163.com (M.W.); gaocl890907@163.com (C.G.); gaobingtao@126.com (B.G.); taojing1029@hotmail.com (J.T.); 2 Sino-France Joint Laboratory for Invasive Forest Pests in Eurasia, Beijing Forestry University, Beijing 100083, China; 3 Department of Forest Protection, College of Forestry, Hebei Agricultural University, Baoding 071033, China; 4 Guangdong Key Laboratory of Animal Conservation and Resource Utilization, Guangdong Public Laboratory of Wild Animal Conservation and Utilization, Institute of Zoology, Guangdong Academy of Sciences, Guangzhou 510260, China; 5 Guangdong Provincial Key Laboratory of Silviculture, Protection and Utilization, Guangdong Academy of Forestry, Guangzhou 510520, China; 6 Research Institute of Tropical Forestry, Chinese Academy of Forestry, Guangzhou 510520, China

**Keywords:** *Sirex noctilio*, biological invasion, gut microbiome, community assembly, evolutionary adaptations

## Abstract

The mutualistic symbiosis relationship between the gut microbiome and their insect hosts has attracted much scientific attention. The native woodwasp, *Sirex nitobei,* and the invasive European woodwasp, *Sirex noctilio*, are two pests that infest pines in northeastern China. Following its encounter with the native species, however, there is a lack of research on whether the gut microbiome of *S. noctilio* changed, what causes contributed to these alterations, and whether these changes were more conducive to invasive colonization. We used high-throughput and metatranscriptomic sequencing to investigate *S. noctilio* larval gut and frass from four sites where only *S. noctilio* and both two *Sirex* species and investigated the effects of environmental factors, biological interactions, and ecological processes on *S. noctilio* gut microbial community assembly. Amplicon sequencing of two *Sirex* species revealed differential patterns of bacterial and fungal composition and functional prediction. *S. noctilio* larval gut bacterial and fungal diversity was essentially higher in coexistence sites than in separate existence sites, and most of the larval gut bacterial and fungal community functional predictions were significantly different as well. Moreover, temperature and precipitation positively correlate with most of the highly abundant bacterial and fungal genera. Source-tracking analysis showed that *S. noctilio* larvae at coexistence sites remain dependent on adult gut transmission (vertical transmission) or recruitment to frass (horizontal transmission). Meanwhile, stochastic processes of drift and dispersal limitation also have important impacts on the assembly of *S. noctilio* larval gut microbiome, especially at coexistence sites. In summary, our results reveal the potential role of changes in *S. noctilio* larval gut microbiome in the successful colonization and better adaptation of the environment.

## 1. Introduction

Transcontinental movement during human trade and travel is resulting in increased rates of biological invasions [1]. The frequency of invasions of numerous insects into new niches is increasing worldwide, which has caused forest ecosystem damage and economic losses [2,3]. The introduction of invasive species is often accompanied by associated microorganisms, and together, they form a unit made up of the hosts and microbes with the native biota [4]. Insects are colonized by different microorganisms, which are often beneficial or required by the hosts [5]. Also, insect guts are inhabited by a wide variety of microorganisms, and they potentially have a mutually beneficial relationship with their hosts [6]. For example, many of the microbes in termites are exclusively found in guts [7]. Digestive symbioses appear most common among insects that feed on wood or other highly lignified plant materials [8,9]. The extended functional traits provided by symbiotic microbes contribute to the competitiveness and fitness of their invasive hosts. The gut microbiota enables invasive species to invade new niches [10]. Therefore, it is of great significance to study the coevolutionary relationship between gut microbes and their hosts to unravel the underlying mechanisms related to invasion.

The European woodwasp, *Sirex noctilio* Fabricius, a wood-boring insect that damages *Pinus* species in its invaded areas, is a major invasive quarantine pest that impacts the local economy and ecosystem [4,11]. It is the only known woodwasp species that could kill living trees by overcoming their self-defenses [12]. Unlike most wood-boring insects, female adults lay eggs directly in the xylem, which is a tissue characterized by very low nitrogen content and high lignification, while others lay eggs in the phloem or in the early stages of larval development [13,14]. In addition, it also injects its symbiotic fungus *Amylostereum areolatum* and toxins (secreted by its venom gland) into the host tree when the female lays eggs [15]. Hence, it is a cooperative mechanism to weaken the hosts. *S*. *noctilio* was first identified in Heilongjiang Province, in northeastern China, and their establishment caused wide-scale death to *Pinus sylvestris* var. *mongolica* [16,17]. So far, it has invaded 24 regions in China, thereby endangering Mongolian pine plantations [18].

The woodwasp, *Sirex nitobei*, is native to Asia with a Palaearctic and Oriental distribution range and mainly attacks *Larix* and *Pinus* species. It has been distributed within some Asian countries where it had not been previously recorded. Similar to *S. noctilio*, the damage caused by a combination of larvae, obligate mutualistic fungus, and toxins weaken host trees. It also attacks pine plants in the nursery. Furthermore, some studies predicted that *S. nitobei* has a high suitability area in China and has the potential to spread to previously unsuitable or moderately suitable areas; current control options for the two species are limited because of their feeding habits [19]. *S. noctilio* has invaded areas where the native species *S. nitobei* were originally distributed, such as the Inner Mongolia Autonomous Region and Jilin Province. Together, the two species have severely affected the timber industry, as most of the hosts are important wood species for coniferous timber production.

A growing number of studies have cataloged and characterized microbial communities by molecular methods [20,21], and variability in the gut microbiome may exist in different species of the same host or different geographic populations of the same species. Huang et al. [22] compare the gut bacterial communities in *Reticulitermes flavipes* termites fed on different plant diets, and gut bacterial richness and diversity was reduced on the corn stover diet, which may have been related to the lower degradation of this diet. Berasategui et al. [8] demonstrated that the gut bacterial community in *Hylobius abietis* was similar at family and genus levels in Europe and was very similar to that of bark beetles, *Ips typography,* that also exploit conifers as a food source. Characterization of the gut microbiota in the domesticated silkworm, *Bombyx mori* and its wild relatives *Acronicta major* and *Diaphania pyloalis*, revealed a highly diverse community, and environmental factors, including diet and human manipulation (egg production), likely influenced this composition [23]. Amplicon sequencing of 46 beetle species showed that the gut bacterial and fungal communities differed among all niches and all food habits of their hosts (carnivores, herbivores, omnivores, and scavengers), except for the fungal communities between carnivores and scavengers [24]. Jones et al. [25] demonstrated that *Spodoptera frugiperda* midgut bacterial communities differed from those of *Helicoverpa zea* collected from the same host plant species at the same site. However, *H. zea* bacterial communities differed between collection sites. Gut microbial community analysis of *Leptinotarsa decemlineata*, collected from nine geographic locations in China, indicated that order and genus were appropriate taxonomic levels to distinguish the geographical sources [26]. 

Environmental factors and host genetics shape the gut microbiota in various animal taxa [27,28]. In insects, the microbial communities vary with geographical locations, and they contribute to adaptation to the local niche [26]. Stochastic and deterministic processes also shape the microbiome [29]. However, it is unknown to what degree each of these variables contributes to the composition of the microbiome in woodwasps. In our previous research, we revealed the larval gut structure and gut microbiota at different developmental stages of *S. noctilio* [13]. To establish an ecological and comparative baseline for experimental studies on the woodwasp gut microbiome, we conducted an investigation on the microbial communities associated with the invasive *S. noctilio* and native *S. nitobei.* Samples were collected from different sites in northeastern China, including the larval guts and their niche, using high-throughput sequencing. In addition, we applied metatranscriptomics to identify the main active group and the predominant activities performed by a specific community. These systematic and comparative analyses provide a foundation for the comprehensive investigation into the gut microbiome of *S. noctilio* and reveal the role of gut microbial symbionts in the unique cooperative degradation mechanism (wood-borer, symbiotic fungus, and gut microbiome) in this insect.

## 2. Results

### 2.1. Microbial Communities Associated with Invasive and Native Woodwasp Species

**Figure 1:**
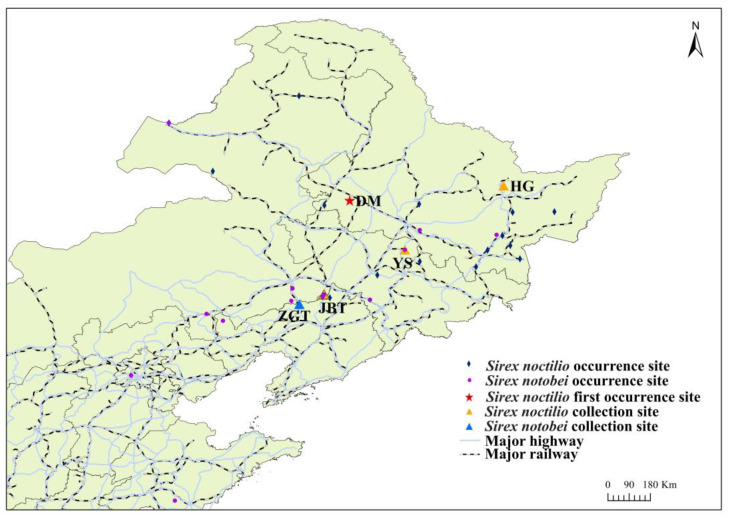
Sampling sites of *Sirex noctilio* and *Sirex nitobei* in northeastern China. Occurrence sites contain the collection sites where DM and HG are *S. noctilio* alone, and YS, JBT, and ZGT are coexistence sites for both two *Sirex* species, *S. noctilio* were collected from DM, HG, YS, and JBT, and *S. nitobei* was collected from ZGT. This figure plotted the sampling distributions merged with those of Sun et al. [18].

We analyzed the bacterial and fungal community composition of the larval gut and frass (frass is originally used as a combination of host xylem and insect feces) of 375 individual woodwasps (*S. noctilio* and *S. nitobei*) inhabiting five different sampling spots (Figure 1), including Duerbert Mongolian Autonomous County, Daqing City, Heilongjiang Province (DM), Hegang City, Heilongjiang Province (HG), Jinbaotun Town, Tongliao City, Inner Mongolia Autonomous Region (JBT), Yushu City, Jilin Province (YS), and Zhanggutai Town, Zhangwu County, Fuxin City, Liaoning Province (ZGT). Overall, 2,191,748 raw 16S rRNA and 2,381,472 ITS2 gene sequence reads were obtained from the larval gut and frass. After quality control and filtering, we obtained 1,485,732 and 2,285,124 high-quality sequence reads, from which 4912 and 1432 OTUs were identified, respectively (see details Table S1). To determine whether the sampling depth was sufficient, OTU-level rarefaction curves based on the Shannon and Sobs indices were generated to compare the richness and evenness among samples (Figure S1), and Shannon, Simpson, ACE, Chao, and coverage indices were estimated as alpha diversity indicators (Table S2), indicating that the vast majority of species in the gut and frass were included in this study.

#### 2.1.1. Different Composition of the Gut Microbiome in the Two *Sirex* Species

**Figure 2:**
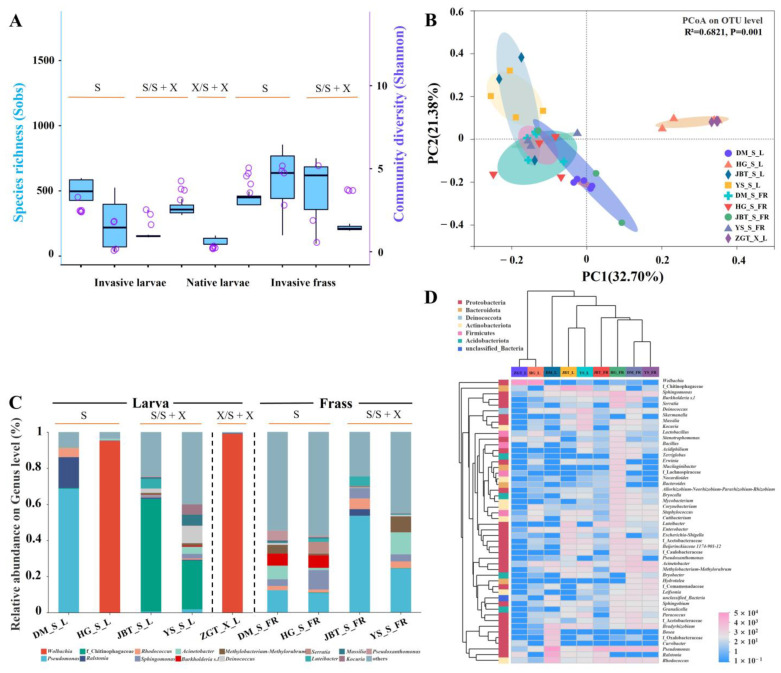
Bacterial community of larval gut and frass between *Sirex noctilio* and *Sirex nitobei*. (**A**) Boxplot of species richness (number of OTUs) and community diversity measured by Sobs (blue) and Shannon (purple) index (one-way ANOVA, LSD post hoc test, *p <* 0.05, see Table S3). (**B**) PCoA plot based on weighted UniFrac distance. Each symbol represents a sample, variation in communities segregated according to host phylogeny, with *S. noctilio* and *S. nitobei* forming discrete groups (PERMANOVA test with 999 permutations, *p* ≤ 0.05, see Figure S2A,B based on Bray–Curtis and unweighted UniFrac distance). (**C**) Relative abundance of bacterial genera in different samples (invasive woodwasp larval gut and frass, native woodwasp larval gut). OTUs that were <5% of average relative abundance in groups were summarized as “others”. (**D**) Heatmap of *Sirex noctilio* and *Sirex nitobei* dominant larval gut and frass bacteria in different regions at the genus level. Relative abundances of the 50 most abundant genera are shown with cluster analysis using Bray–Curtis distance, followed by an average–linkage method. (Species abbreviations, S: *S. noctilio* in separate existence sites, including DM and HG; S/S + X: *S. noctilio* in coexistence sites, including JBT and YS; X/S + X: *S. nitobei* in coexistence sites, including ZGT; L, larva; FR, frass.).

**Figure 3:**
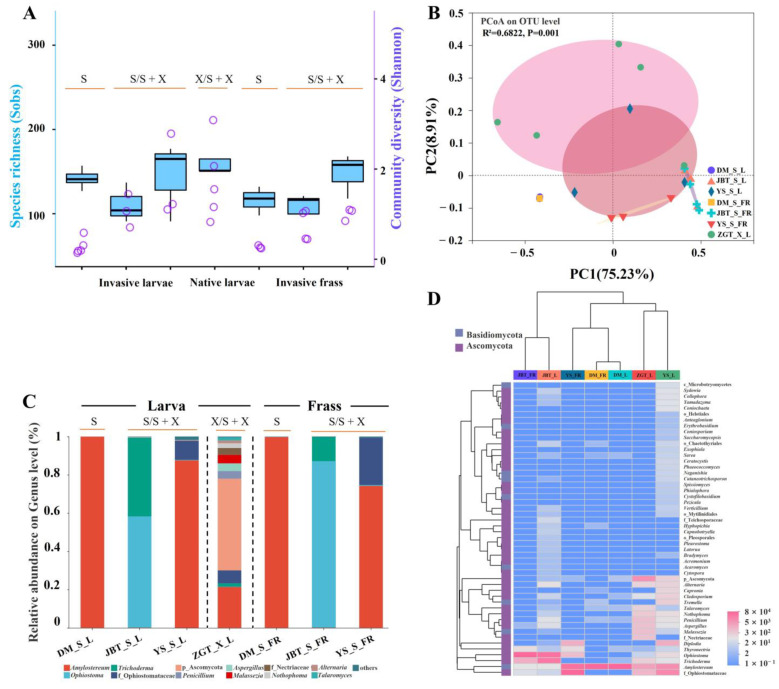
Fungal community of larval gut and frass between *Sirex noctilio* and *Sirex nitobei*. (**A**) Boxplot of species richness (number of OTUs) and community diversity measured by Sobs (blue) and Shannon (purple) index (one-way ANOVA, LSD post hoc test, *p <* 0.05, see Table S3). (**B**) PCoA plot based on weighted UniFrac distance. Each symbol represents a sample, variation in communities segregated according to host phylogeny, with *S. noctilio* and *S. nitobei* forming discrete groups (PERMANOVA test with 999 permutations, *p* ≤ 0.05, see Figure S2C,D based on Bray–Curtis and unweighted UniFrac distance). (**C**) Relative abundance of fungal genera in different samples (invasive woodwasp larval gut and frass, native woodwasp larval gut). OTUs that were <1% of average relative abundance in groups were summarized as “others”. (**D**) Heatmap of *Sirex noctilio* and *Sirex nitobei* dominant larval gut and frass fungi in different regions at the genus level. Relative abundances of the 50 most abundant genera are shown with cluster analysis using Bray–Curtis distance, followed by an average-linkage method. (Species abbreviations, S: *S. noctilio* in separate existence sites, including DM and HG; S/S + X: *S. noctilio* in coexistence sites, including JBT and YS; X/S + X: *S. nitobei* in coexistence sites, including ZGT; L, larva; FR, frass.).

Alpha diversity comparisons of the bacterial communities revealed that *S. nitobei* in ZGT had lower levels of larval gut microbial diversity (Shannon index) and richness (sobs index) than *S. noctilio* (Figure 2A and Table S2). However, *S. nitobei* showed greater larval gut microbial diversity (Shannon index) and richness (Sobs index) for fungal communities than *S. noctilio* (Figure 3A and Table S2).

PCoA based on the Bray–Curtis (Figure S2A,C) and unweighted UniFrac (Figure S2B,D) matrices were used to evaluate the structure of the microbiome in the two woodwasp species, indicating that the bacterial and fungal communities were able to separate the *S. noctilio* from *S. nitobei* (*p =* 0.001). However, based on weighted UniFrac matrices, the bacterial communities in *S. noctilio* in HG were slightly similar to *S. nitobei* (*p =* 0.001) (Figure 2B).

Microbial community composition also revealed the differences between the two *Sirex* species. The most prevalent bacterial phylum was Proteobacteria, which, together with Bacteroidetes, Actinobacteria, Firmicutes, and Acidobacteria, was detected in all samples (Figure S3), and the major fungal phyla were Ascomycota and Basidiomycota (Figure S5). At the genus level, a total of 1154 bacterial genera and 188 fungal genera belonging to 44 and 6 phyla were detected in the five sites, respectively (Table S1). Compared to its native relatives, *S. nitobei*, *Wolbachia* (99.0%) was the highest relative abundant bacterial genera (Figure 2C). Aside from unclassified P_Ascomycota (47.9%), *Amylostereum* (21.4%) was the major fungal genera (Figure 3C). The distribution of different OTUs in host species showed that the samples are clustered based on host phylogeny (Figure 2D and Figure 3D). Furthermore, LEfSe analysis showed that *Pseudomonas, Luteibacter,* and *Enterobacter* (all in the Gammaproteobacteria class) were significantly more abundant in *S. noctilio* than in *S. nitobei*, whereas *Wolbachia* (Alphaproteobacteria) was significantly more abundant in *S. nitobei* at the genus level (Figure S7A). Among fungal communities, *Tremella* and *Aspergillus* were significantly more abundant in *S. noctilio* and *S. nitobei* at the genus level, respectively (Figure S7B).

#### 2.1.2. Composition of the *S. noctilio* Gut Microbiome in Different Geographic Locations

*Sirex noctilio* showed the greatest diversity of bacterial OTUs in DM and the lowest in HG (Figure 2A and Table S2). The region between YS and JBT were similar (Figure 2B); *Pseudomonas* (68.6%) dominated in DM, unclassified f_Chitinophagaceae dominated in JBT (62.4%) and YS (27.1%), *Wolbachia* (95.3%) dominated in HG, and a highly diverse gut microbiota was found in JBT and YS (Figure 2C). 

It had the lowest diversity but higher richness of fungal OTUs in DM but higher diversity and richness in YS (Figure 3A and Table S2). Most gut and frass OTUs from different regions were clustered together (Figure 3B); *Amylostereum* (99.9%) dominated in DM, *Ophiostoma* (58.2%) and *Trichoderma* (41.1%) dominated in JBT, unclassified f_Ophiostomataceae (49.3%) and *Amylostereum* (34.7%) dominated in YS, and highly diverse gut microbiota was found in JBT and YS, which was similar to the bacterial community (Figure 3C). 

The relative abundance at the different taxonomic levels, from major bacterial and fungal phylum to genus, was different within the different groups (Figures S3 and S5, Tables S4 and S5). Overall, 57 bacterial genera (7.36%) were shared among all larval gut groups regardless of species (Figure S4A), and 17 fungal genera (9.83%) were shared among all larval gut groups (Figure S6A).

#### 2.1.3. *Sirex noctilio* Gut Microbiome Are Different from Frass

Overall, the richness and diversity of frass bacterial communities were higher than those of larval gut (Figure 2A and Table S2). However, the richness and diversity of frass fungal communities were lower than those of larval gut (Figure 3A and Table S2). The difference between the larval gut and frass bacterial communities was significant (*p =* 0.002) (Figure S2E); however, it was not significant for fungal communities (*p =* 0.428) (Figure S2F).

**Figure 4:**
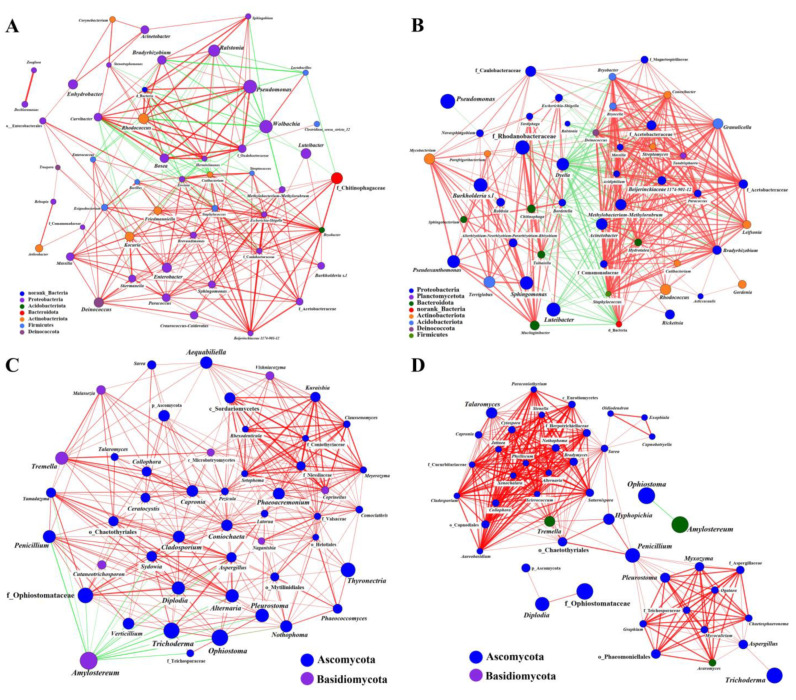
Co-occurrence analysis of *Sirex noctilio* gut (**A**,**C**) and frass (**B**,**D**) microbiota co-occurring bacteria (**A**,**B**) and fungi (**C**,**D**) at the genus level in different regions. Size and color of the nodes represent relative abundance of the microbiota and heritability estimates, respectively. Solid lines in red and in green denote positive and negative correlations, respectively. The width reflects the strength of the correlation.

Similar patterns of bacterial clustering were revealed by co-occurrence analysis of the microbiome in the larval gut and the frass, although the relationships’ strengths varied (Figure 4A,B). Analysis of the relative abundance of the top 50 bacterial genera identified 219 positive and 51 negative correlations in the larval gut, while the larval frass had 363 positive and 109 negative correlations (Table S6). For fungi, co-occurrence analysis of larval gut and frass fungal communities showed different patterns, especially of the major genera (such as *Amylostereum* and *Ophiostoma*). There were 346 positive and 16 negative correlations in the larval gut, while the larval frass had 305 positive and 23 negative correlations (Table S7). A network analysis of the bacterial community found that larval frass had a significantly higher degree and closeness centrality than those of larval gut, while larval gut had slightly higher betweenness connectivity (Figure S8A,B). Interestingly, the results were reversed in the fungal network analysis (Figure S8C,D). In general, frass variation was far less than gut variation; even the bacterial community of frass was similar at four sites, which explained to some extent that the larvae recruit different genera from their surroundings’ environmental microbiomes according to their growth and developmental needs. 

### 2.2. Potential Factors Affecting Microbial Communities

#### 2.2.1. Effects of Meteorological Factors on *S. noctilio* Gut Microbiome

**Figure 5:**
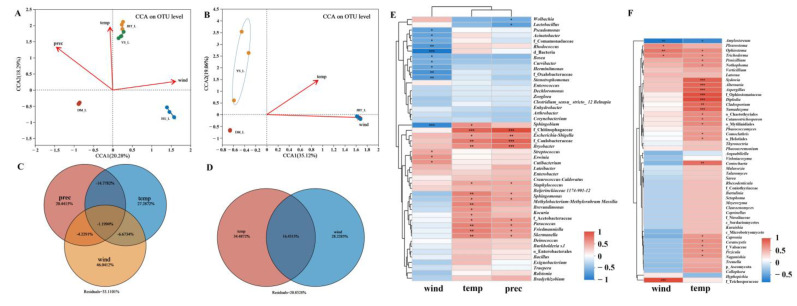
Relationship between climate factors and *Sirex noctilio* gut bacterial (**A**,**C**,**E**) and fungal (**B**,**D**,**F**) communities. (**A**,**B**) Relationships between microbial communities and climatic factors (CCA). Only significant factors are shown in the figure; the direction of the arrows in the figure indicates positive/negative correlations, and the angle between the explanatory and response variables reflects the correlation coefficient. (**C**,**D**) VPA results showing the relative explanatory rates of climatic factors on bacterial and fungal community variation. Climatic factors include near-surface wind speed (wind), precipitation rate (prec), and near-surface air temperature (temp). (**E**,**F**) Heat map of the top 50 genera in abundance and climate factors: red indicates positive correlation, blue indicates negative correlation, * 0.01 < *p* ≤ 0.05, ** 0.001 < *p* ≤ 0.01, *** *p* ≤ 0.001.

Climate factors screened by VIF analysis showed that near-surface wind speed (wind), precipitation rate (prec), and near-surface air temperature (temp) may be statistically related to the differences observed in both bacterial and fungal community compositions (Figure 5 and Figure S9). CCA analysis assessed the effect of climate factors on the composition of *S. noctilio* gut microbiome, with the first two axes explaining 38.57% and 54.18% of the total variation in the structural composition of bacterial and fungal communities, respectively (Figure 5A,B). The PERMANOVA test revealed that wind (R^2^ = 0.6627, *p* = 0.008 and R^2^ = 0.9987, *p* = 0.008), temp (R^2^ = 0.9395, *p* = 0.001 and R^2^ = 0.8235, *p* = 0.006) and prec (R^2^ = 0.9559, *p* = 0.001) significantly related with the bacterial and fungal communities (Table S8). Furthermore, VPA was used to determine the relative contribution of each climatic factor to *S. noctilio* gut microbiome; wind, temp, and prec largely contributed to the changes in the gut bacterial community (46.04%, 27.29%, and 20.44%) and fungal community (28.23% and 34.49%), respectively (Figure 5C,D). Spearman correlation analysis of highly abundant genera with climate factors, for bacteria, showed that temp and prec had a significant positive correlation with the highest abundant genera, such as f_Chitinophagaceae, f__Caulobacteraceae, and *Bryobacter*, while wind showed a significant negative correlation with the most highly abundant genera, such as *Sphingobium*; for fungi, temp and wind were significantly positively correlated with the most highly abundant genera, such as *Alternaria*, *Aspergillus,* and *Cladosporium*, while both were significantly negatively correlated with *Amylostereum* (Figure 5E,F). 

#### 2.2.2. Coexistence of the Two *Sirex* Niches Enriched *S. noctilio* Gut Microbiome

**Figure 6:**
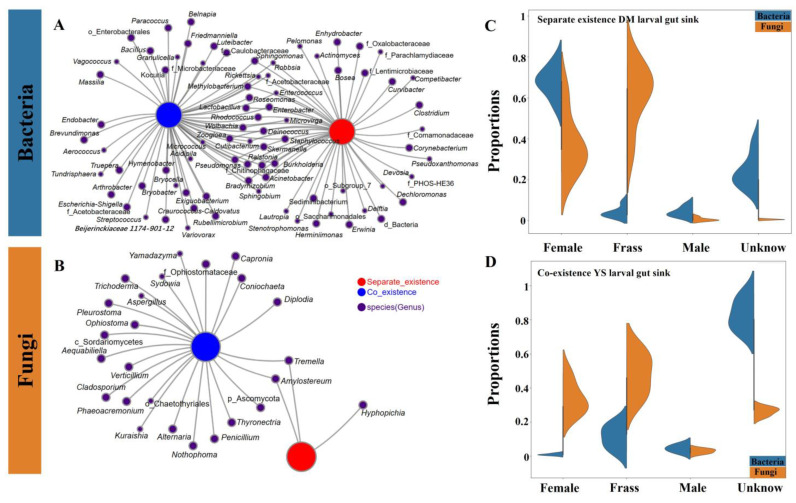

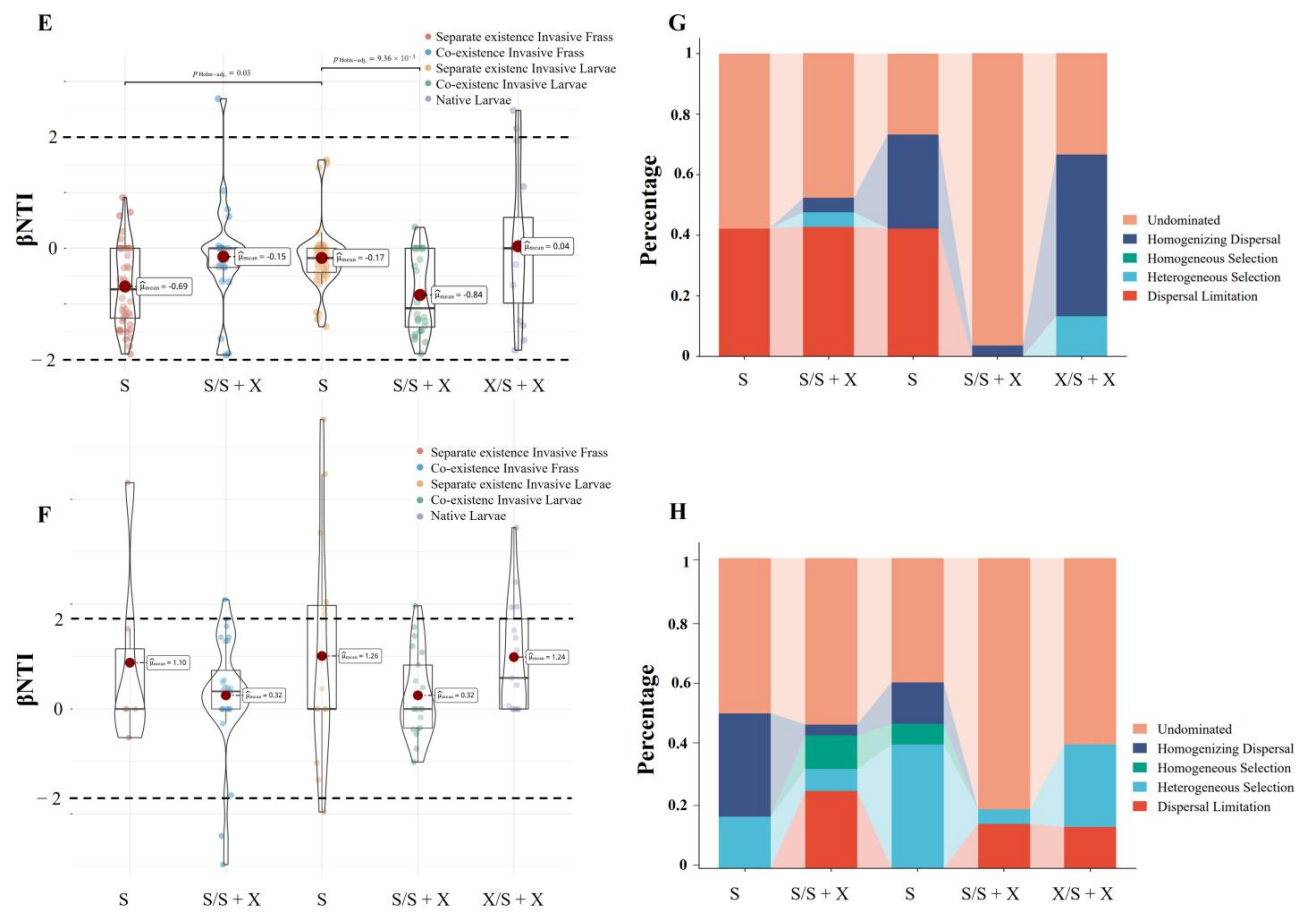
Co-occurrence networks (**A**,**B**), source tracking (**C**,**D**), and ecological processes analysis of *Sirex noctilio* gut bacterial and fungal communities in the separate existence and coexistence sites. (**A**,**B**) Networks show co-occurrence relationship of genera in coexistence and separate existence sites samples, and only genera with abundance (number of sequences) greater than 50 are displayed. (**C**,**D**) Source-tracking analysis graph that represents predictions of sources, with colored violin plots representing the proportion of each source in a sample. Unknown indicates classification of unknown sources. Box and whisker plots of contributions of deterministic (|βNTI| ≥ 2) and stochastic processes (|βNTI| < 2) on bacterial (**E**) and fungal (**F**) community assembly in each group. The relative contributions of ecological processes in driving the bacterial (**G**) and fungal (**H**) assembly in each group. (Species abbreviations, S: *S. noctilio* in separate existence sites, including DM and HG; S/S + X: *S. noctilio* in coexistence sites, including JBT and YS; X/S + X: *S. nitobei* in coexistence sites, including ZGT; L, larva; FR, frass.).

In total, we recovered 531 bacterial and 123 fungal genera nodes from coexistence site samples and 518 bacterial and 53 fungal genera nodes from separate existence site samples. There were more genera at the coexistence site than at the separate existence site, as indicated by the network (Figure 6A,B) and circle diagram (Figure S10), with differences in both the dominant genus and its relative abundance (Tables S9 and S10). Notably, this was also similar to the larval gut microbial community in the previously analyzed coexistence sites (YS and JBT) that showed significantly higher α-diversity and community composition compared to the separating existence sites (HG and DM) (Figure 2 and Figure 3). 

Analysis of the source of gut microbes was conducted to better understand the influence of frass and adult gut microbiome on larval gut microbiome. The result revealed that, in the separate existence site, the sources of *S. noctilio* larval gut bacteria communities were mainly from the female gut and unknown, with small amounts from the frass and male gut. The sources of larval gut fungal communities were mainly from the frass and female gut, with very small amounts from the male gut and unknown (Figure 6C). By contrast, the source of *S. noctilio* larval gut bacteria communities in the coexistence site was mainly from the frass and unknown, with small amounts from the male gut and female gut. The source of larval gut fungal communities was mainly from frass, female gut, and unknown, with a very small amount from the male gut (Figure 6D). 

To further quantify the relative contributions of different ecological processes to the two *Sirex* species before and after their encounter, a null model based on βNTI and RC_bray_ was used (Figure S11). The results showed that bacterial and fungal community assembly in all the different types of samples (frass, invasive larvae, and native larvae) was primarily driven by stochastic processes (|βNTI| < 2) (Figure 6E,F). Dispersal and undominated (e.g., drift, diversification, weak dispersal, and weak selection) were the main factors driving both bacterial and fungal communities in *S. noctilio* larval gut. However, before encounters, dispersal (dispersal limitation and homogeneous dispersal) had a higher relative contribution to the assembly of *S. noctilio* larval gut bacterial communities, while undominated factors were prominent after encountering (Figure 6G). Deterministic and stochastic processes drove the construction of the *S. noctilio* larval gut fungal community, with both deterministic (homogeneous and heterogeneous selection) and stochastic (undominated and homogeneous dispersal) processes playing a certain role prior to the encounter of the two *Sirex* species. However, after the encounter, they were more dominated by stochastic (dispersal limitation and undominated) processes (Figure 6H), which is also similar to the results on the source tracking (Figure S12). 

### 2.3. Functional Differences between Woodwasp Larval Gut and Frass

#### 2.3.1. Functional Prediction Based on Amplifier Sequencing

**Figure 7:**
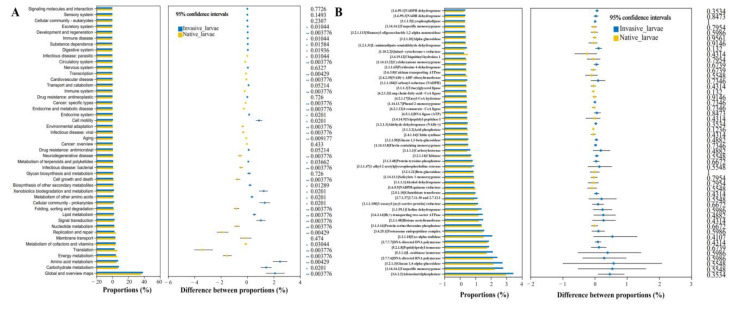

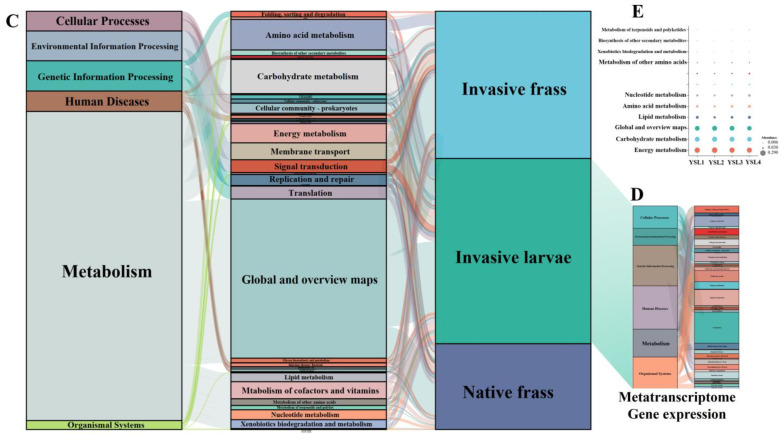
Functional prediction based on amplifier sequencing (**A**–**C**) and metatranscriptome gene expression (**D**,**E**). (**A**–**C**) PICRUSt2-identified functional differences in the gut microbiome between the two species, assessed using Wilcoxon rank sum test for gut bacterial communities (**A**), heatmap of enzymes annotation for gut fungal communities (**B**), and average relative abundance of 16S rRNA-annotated KEGG level 2 pathway in the Sankey diagram (**C**), * 0.01 < *p* ≤ 0.05, ** 0.001 < *p* ≤ 0.01; (**D**,**E**) Average relative abundance of metatranscriptom-annotated KEGG level 2 pathway in the Sankey diagram (**D**), and particularly genes were expressed in the metabolism-related pathway (**E**). The relatively low proportion of categories that were still unclear in Figure 7C,D was shown in Supplementary Table S12.

For bacterial communities, a total of 7736 genes were classified into 6 level-1 pathways and 46 level-2 pathways based on the KEGG database. Of these 46 pathways, most gene functions were related to metabolism (74.6% and 68.9%), environmental information processing (7.8% and 5.9%), and genetic information processing (5.9% and 13.2%) in *S. noctilio* and *S. nitobei* larval gut, respectively (Figure 7C). We found that 36 KEGG pathways were significantly differentially enriched (Wilcoxon’s t, *p* value < 0.05) (Figure 7A). All metabolism pathways were significantly different within the two species. Gene families in the following categories were enriched in *S. noctilio*: carbohydrate metabolism, amino acid metabolism, lipid metabolism, xenobiotic biodegradation and metabolism, metabolism of other amino acids, biosynthesis of other secondary metabolites, glycan biosynthesis and metabolism, and metabolism of terpenoids and polyketides. By contrast, gene families in the following categories were enriched in *S. nitobei*: energy metabolism, metabolism of cofactors and vitamins, replication and repair, and nucleotide metabolism. In addition, only one KEGG pathway was significantly differentially enriched between *S. noctilio* larvae and frass (Figure S13C). The coexistence sites for *S. noctilio* larval gut bacterial communities were significantly differentially enriched in metabolism, such as carbohydrate metabolism, biosynthesis of other secondary metabolites, glycan biosynthesis and metabolism, and metabolism of terpenoids and polyketides, whilst the separate existence sites were significantly differentially enriched in environmental information processing (Figure S13D). Meanwhile, the coexistence sites and separate existence sites for frass bacterial communities were not significantly differentially enriched (Figure S13E), which was consistent with their amplicon taxonomic profiles (Figure 2C). 

For fungal communities, a total of 867 enzymes were annotated for the two species based on the KEGG database. The relative abundance of the top 50 enzymes was not significantly differentially enriched (Figure 7B). Similarly, there was no significantly different enrichment between *S. noctilio* larvae and frass (Figure S13F). The separate existence sites for *S. noctilio* larval gut fungal communities were significantly differentially enriched in Glucan 1,4-alpha-glucosidase EC (3.2.1.3), Exo-alpha-sialidase EC (3.2.1.18), Carboxylesterase EC (3.1.1.1), Glucan 1,3-beta-glucosidase EC (3.2.1.58), Chitin synthase EC (2.4.1.16), and Cytochrome-c oxidase EC (1.9.3.1), whilst the coexistence sites were significantly differentially enriched in Beta-glucosidase EC (3.2.1.21), Alpha-glucosidase EC (3.2.1.20), and Mannosyl-oligosaccharide 1,2-alpha-mannosidase EC (3.2.1.113) (Figure S13G), which is associated with lignocellulose degradation. Meanwhile, for frass fungal communities, the coexistence sites and separate existence sites were not significantly differentially enriched (Figure S13H), which was consistent with their amplicon taxonomic profiles (Figure 3C).

Functional prediction showed that most of the enzymes were associated with the maintenance of life activities. There were also many genes potentially related to lignocellulose degradation and nitrogen fixation (Figure S13A), which was consistent with their metatranscriptome profiles (Figure S13B). The detailed number of predicted gene families has been provided in Supplementary Table S11. Most of the predicted genes are involved in the lignocellulose degradation pathways, including glucan 1,4-alpha-glucosidase (EC3.2.1.3), carboxylesterase (EC3.1.1.1), and beta-glucosidase (EC3.2.1.21). For lignin degradation, laccase (EC1.10.3.2) was the most abundant. For nitrogen fixation, we predicted key genes for nitrogenase component proteins (*nifH*), i.e., nitrogenase (EC1.18.6.1). 

#### 2.3.2. Microbial Community Composition and CAZymes Identified in the Metatranscriptomes of the *S. noctilio* Larval Gut Microbiome

Total RNA was extracted from 40 larval guts of *S. noctilio* and collected in YS. After sequencing the samples, a total of 587,885 sequence reads from four libraries were generated from the gut metatranscriptome. The assembly result is shown in Table S13. These datasets, which represent composites of transcripts from the host and the microbial community, were pooled to generate a single overall assembly consisting of 28,816 contigs (Table S14). The general taxonomic assignment from the annotated sequences showed that most of the annotations belonged to the phyla Arthropoda, Nematoda, Proteobacteria, and Chordata (Table S15). A total of 23,180 contigs were classified as belonging to the class insect, especially Hymenoptera (or 79% of the total 22,751 contigs). The remaining nonhost fraction related to the microbial community was 467 contigs (or 1.6%). Hence, our analysis of metabolic pathways focused on both the host and the microbial community, which showed that the identified communities were of the less abundant group, such as Streptophyta, Firmicutes, Ascomycota, Actinobacteria, Glomeromycota, and Cyanobacteria, except the phyla Proteobacteria (see Table S16 for details).

Sequence homology-based taxonomic assignment of functional genes from *S. noctilio* larval gut metatranscriptome assemblies indicated some differences between the transcript distribution among KEGG level-2 categories and the prediction of 16S rRNA function (Figure 7C,D), which may be related to the instantaneous state of the sampling. Among the metabolism, the most highly expressed functional categories in the *S. noctilio* larval gut microbiota included energy metabolism, carbohydrate metabolism, lipid metabolism, and amino acid metabolism (Figure 7E). We used the CAZy database to identify carbohydrate-active enzymes in the metatranscriptome, which results showed that *S. noctilio* larval gut and larval gut microbiome collectively produced 99 genes to partial or complete glycoside hydrolases (GHs), 173 glycoside transferases (GTs), 127 carbohydrate-binding modules (CBMs), 46 carbohydrate esterases (CEs), 13 auxiliary activities (AAs), and 2 polysaccharide lyases (PLs) (Table 1). To determine the origin of these key genes associated with lignocellulose catabolism, we examined their taxonomic composition. Notably, NR annotations of almost all unigenes were identified as insect origin, with only two unigenes annotated as bacterial origin, suggesting that *S. noctilio* larval gut was also equipped to degrade lignocellulose.

## 3. Discussion

This study represents the first large-scale study investigating both the bacterial and fungal communities associated with the invasive woodwasp *S. noctilio* sampled at different locations and the native woodwasp *S. nitobei*, as well as the metatranscriptome of *S. noctilio* larvae gut. The results showed that the coexistence of the invasive woodwasp *S. noctilio* and native *S. nitobei* profoundly affected and remodeled the assembly and metabolic functions of the *S. noctilio* larval gut microbiome. 

Interestingly, *S. noctilio* was distributed along the major highways and railways in China (Figure 1), showing that the impact of human activities (such as wood packaging and timber transportation, etc.) on the spread of woodwasp populations cannot be ignored. Simultaneously, the expansion of international trade has facilitated the invasions of numerous insects and pathogens into new regions [3]. Under future climate change scenarios, the species is likely to spread widely as more areas become more suitable and habitable through favorable environmental conditions [19,30,31]. 

Insect gut microbiota is shaped by a range of complex biotic and abiotic factors such as developmental stages, host plants, geographic sources, environmental microbes, temperature, humidity, etc. [26,27,29]. It was reported that host species dominate over geographical sites in shaping honeybee gut bacterial communities [32]. Coincidentally, our results revealed that the two *Sirex* species harbor a very diverse microbiota, although both populations were collected from *Pinus sylvestris* var. *mongolica*. *Wolbachia*, a common symbiont in insects, is well known for manipulating host reproduction via various phenotypic effects [33], especially in a few parthenogenetic insects [34]. Therefore, we hypothesize that this could be the cause of the native species’ low population level and the lack of research carried out on *S. nitobei* prior to *S. noctilio*’s invasion. Likewise, even less research has been carried out on other native *Sirex* species abroad [35]. There were 66 fungal OTUs detected in *S. juvencus* adult females, and unidentified sp. 5671_109, *A. chailletii*, and *Penicillium angulare* showed a higher relative abundance in *S. juvencus* [36]. Functional prediction of the larval gut microbiome showed a significant difference between the bacterial community in the two *Sirex* species. However, there was no difference in the fungal community, as *Amylostereum* was still the dominant genus. The complex bacterial structure may enhance the adaptation of *S. noctilio* to new niches more rapidly and flexibly. Nonetheless, since we sampled only one area of *S. nitobei*, the differences in the gut microbial community composition and function predicted of the two species may be related to their fewer samples, and we should subsequently increase the number of samples taken from the native species as much as possible before comparing them. In our previous study, we only compared the different developmental stages of *S. noctilio* in DM (i.e., the area where only *S. noctilio* was distributed) to illustrate how it adapts to host xylem [13], while this study focuses more on the differences in the composition and predicted function of *S. noctilio* larval gut microbiome and frass from different sites, and explores more about how the complex gut microbiome has been changed after the invasion of *Sirex noctilio* into China so as to be more conducive to its stable colonization and dispersal to get along with the native species *S. nitobei* better. In addition, by assembling and annotating *Mycocepurus goeldii* and *Atta sexdens rubropilosa* metagenomes [37], the microbiota associated with fungus-growing insects presents a distinctive taxonomic profile, dominated by Gammaproteobacteria at the class level. The authors suggest that the microbiota could be functionally adapted to the fungiculture environment, which is also similar to our results, where the larval gut bacterial community was more complex and diverse, while the fungal community structure is relatively simple.

The diversity of the *S. noctilio* larval gut microbiome differed significantly across four sites, indicating that factors other than host specificity played an important role in the gut microbiome assembly in the invasive species. The results showed that wind, temp, and prec explained the changes in *S. noctilio* larval gut bacterial community and fungal community, and their diversity was higher in JBT and YS than in DM and HG essentially, while YS and JBT were located at lower latitudes. Ge et al. [32] proposed that the higher diversity of *Apis cerana* and *A. mellifera* gut microbial communities at low latitudes may be the result of higher temperatures and precipitation, which tend to accelerate community exchange. At the same time, differences are most often related to dietary differences [38], although they may be mediated by environmental factors. The current environmental factors are the result of correlation analysis and have not established a direct causal relationship. As the gut environment is much more constant, their mediation by environmental influences requires further research.

In addition to the effects of abiotic environmental factors, biotic factors (frass and adult gut microbiome) and the stochastic process of microbial community assembly prior to and after encounters with the native *S. nitobei* also had an impact on the larval gut microbiome in the invasive *S. noctilio*. The female and frass source to the larval gut microbiome was greatly reduced, and that from unknown sources to the larval gut microbiome was increased in the coexistence sites. Both deterministic and stochastic processes played a certain role prior to the encounter of the two *Sirex* species; however, they were more dominated by stochastic processes after the encounter. This suggested that the encounter between the two *Sirex* species may have remodeled the larval gut microbiome. Similar results demonstrating the importance of stochastic processes were also observed in studies on gut microbial communities in *Drosophila simulans* and *Dicranocephalus wallichii bowringi* [39]. Another study also showed that *Curculio chinensis* gut microbiome was derived from the surrounding environmental microbiome and was more affected by the microbiome from soil than that from fruits [40]. However, our results demonstrated that both factors contribute to gut microbiome community assembly, which would perhaps be more conducive to *S. noctilio* spreading colonization.

In terms of functional prediction, *S. noctilio* larval gut microbiome showed significant differences between coexistence and separate existence sites. *S. noctilio* larvae feed on the host xylem for the supplement of sugar, amino acids, and other essential nutrients for their growth and development [13,15]; this may partly explain why the majority of the gut microbiome are involved in carbohydrate metabolism, amino acid metabolism, and lipid metabolism. The extent of functional redundancy in these microbial communities and how this affects measures of diversity and niche overlap is also unclear [41]. This raises a possible question of whether the gut microbiome arises partly through insect functional requirement and selection across insect–microbiome coevolution. Bacterial–fungal interactions are also very important in the ecology of microbial communities. A study revealed the mechanism by which bacterial–fungal transboundary interactions regulate soil organic carbon decomposition [42]. However, the functional relevance of bacteria–fungi interactions in the insect gut, in terms of their physiological consequences for the host, remains unknown, and this may be the focus and direction of future research.

*Amylostereum*, a mutualistic symbiotic fungus of *Sirex* [15], is suggested to be transmitted horizontally from invasive *Sirex* species to native species [4,43]. Meanwhile, for *Amylostereum* genera, our amplicon data indicate that only *A. areolatum* was present in *S. noctilio* larval gut fungal communities, whereas two symbiotic fungi, *A. areolatum* and *A. chailletii*, were present in *S. nitobei* larval gut fungal communities. Similarly, our results showed that the contribution of heterogeneity selection in *S. nitobei* larval gut fungal communities was higher than that in *S. noctilio* in the coexistence sites of the two *Sirex*. This may underlie the differences in the composition of fungal communities. To date, no record has been made of the transfer of *Amylostereum* from the native species *S. nitobei* to *S. noctilio* in China [44]. This implies that *S. noctilio* has its own survival strategy even though it has not acquired other *Amylostereum* genera from native species. In addition to its higher aggressiveness [12], the higher egg-laying numbers [12], the earlier emergence time of adults compared to *S. nitobei* in China, as well as a more complex and varied gut microbiome [45], which may be a more successful survival strategy. Taken together, our study shows how the gut microbiome helps *S. noctilio* to invade and colonize new areas better. 

## 4. Materials and Methods

### 4.1. Sample Collection and Larval Identification

The larvae and frass of *S. noctilio* were collected from northeastern China (DM, HG, JBT, and YS), and *S. nitobei* was collected from ZGT during the May period from 2016 to 2018 (Table 2). We randomly selected the *p. sylvestris* var. *mongolica* (the stem of the tree has holes and resin drops [43]), cut sample pines into wood logs, and brought them back to the quarantine laboratory (Beijing Forestry University). Then, the collection of larvae and frass was carried out using a wood splitter (LS7T-520, Baiduan Industry and Trade Co., Ltd., Shanghai, China) and axe. The identification of larvae (primers see Table S17) and the anatomy of their guts were performed according to our previous work [13], and larvae were surface sterilized before dissection.

### 4.2. Total DNA and RNA Extraction, Library Preparation, and Sequencing

The total nucleic acid was extracted from larval gut and frass samples (5–8 guts/frass for one sample, Table 1), which was ground in liquid nitrogen using sterile pestles. DNA extraction and PCR amplification programs were carried out according to our previous work [13]. Bacteria and fungi were identified with universal primers (Table S17). The 16S rRNA and ITS2 region were amplified in triplicates and mixed with DNA. We included appropriate negative controls at the DNA extraction, and PCR reactions were performed in triplicates, including a negative control without a DNA template and a positive control. Equal volumes were pooled for Illumina MiSeq sequencing (Majorbio, Shanghai, China) according to a standard protocol.

Total RNA was extracted from only four samples (5–8 guts/frass for one sample) collected from Yushu City (YS). Samples were ground in liquid nitrogen and ground using sterile pestles. Thereafter, we utilized the Tissue/Cellular RNA Rapid Extraction Kit (Aidlab Biotechnologies Co., Ltd., BJ, CHN) following the manufacturer’s protocol. The concentration and quality of total RNA produced was estimated with a NanoDrop 2000 spectrophotometer (Thermo Fisher Scientific, Wilmington, DE, USA) and an Agilent 2100 Bioanalyzer (Agilent Technologies, Palo Alto, CA, USA), respectively. Additionally, gel electrophoresis can be used to assess the integrity of the total RNA in the samples. Ribosomal RNA was removed from the total RNA samples using an Epicentre Ribo-zero™ rRNA Removal Kit (Epicentre, Madison, WI, USA). Prior to the construction of libraries using the TruSeq^TM^ RNA Sample Preparation Kits v2 (Illumina, San Diego, CA, USA), three independent RNA extracts of each sample were combined. The barcoded libraries were paired-end sequenced on the Illumina NovaSeq 6000 (Majorbio, Shanghai, China) according to the manufacturer’s instructions.

### 4.3. Amplicon Sequence Data Processing and Statistical Analysis 

Raw sequences were deduplicated, quality-filtered, and merged as described in our previous work; afterward, high-quality reads were analyzed using QIIME v1.9.1 [46] and visualized using R v4.1.2.

Then, reads were clustered into operational taxonomic units (OTUs) at a 97% sequence similarity cutoff using the Uparse pipeline of Usearch v7.0 [47]. Representative sequences for each OTU were classified taxonomically using RDP Classifier v2.2 [48] for bacteria that were compared to the SILVA 138 database [49] and for fungi that were compared to the UNITE v8.0 database [50], with a confidence threshold of 70%. OTUs identified as unclassified bacteria or fungi at the phylum level, archaea, mitochondria, or chloroplasts were excluded. To avoid the affection of sequencing depth in different samples, rarefaction curves were calculated by QIIME using the script “single_rarefaction.py”; sequences from different samples were rarefied to the same depth, including a large portion of the OTUs and diversity. Alpha diversity analysis was calculated for the different groups and visualized using R packages “ggplot2”. Among this, Shannon and Sobs indices were calculated for the different groups, and values were compared using a one-way ANOVA least significant difference (LSD) test. Beta diversity analyses were conducted using QIIME and visualized using R packages “vegan” and “ggplot2”. Principal component analysis (PCoA) was conducted to investigate structural variation in microbial communities, using Adonis based on the Bray–Curtis and the unweighted/weighted UniFrac distance metrics with 999 permutations. The bar plots of the relative abundance of bacteria and fungi were visualized by the R package “barplot” based on the rarefied OTU table. Heat maps for correlation analysis were generated using the R package “vegan” and “ggplot2”, and the Bray–Curtis method was used to calculate the distance, followed by average-linkage clustering to visualize the relationship between samples according to the median. Venn diagram was generated by the R package “VennDiagram”. The linear discriminant analysis effect size (LEfSe) was conducted to test the differences in abundances from phylum to genus among different groups [51]. Also, the linear discriminatory analysis (LDA) was conducted to estimate the effect size for each selected classification. For a genus network-based analysis, network maps were constructed using QIIME and visualized using Cytoscape v3.0.1 [52]. 

Functional predictions were performed using PICRUSt2 v1.1.0 [53]. The PICRUSt2 generates predictions from 16S rRNA and ITS2 data using annotations of sequenced genomes in the Greengene database and KEGG database. Further, these function genes were classified and assigned to the relevant KEGG pathways and enzymes and were compared statistically using nonparametric Kruskal–Wallis tests for pairwise comparisons. 

### 4.4. Metatranscriptome Sequence Data Processing and Statistical Analysis

The 3′ and 5′ ends were stripped using SeqPrep (https://github.com/jstjohn/SeqPrep accessed on 19 March 2016). Low-quality reads (length < 50 bp or Phred score < 20 or having N bases) were removed by Sickle v1.33 (https://github.com/najoshi/sickle accessed on 24 July 2014). Next, we used cross-match to search a database of Illumina adaptor sequences, and adaptor contaminations (http://www.phrap.org/ accessed on 14 April 2015) were filtered. rRNA reads were removed using SortMeRNA v2.1 (https://bioinfo.univ-lille.fr/sortmerna/ accessed on 2 February 2016) [54], aligning with the SILVA 138 database. De bruijn-graph-based assembler SOAP denovo v1.06 was employed to assemble short reads. K-mers, varying from 1/3~2/3 of read length, were tested for each sample. Scaffolds with a length over 300 bp were retained for statistical tests; we evaluated the quality and quantity of scaffolds generated by each assembly and finally chose the best K-mer, which yielded the minimum scaffold number and the maximum value of N50 and N90. Then, scaffolds with a length of over 300 bp were extracted and broken into contigs without gaps. Contigs were used as input for Prodigal v2.6.3 software [55] for further gene prediction and annotation. Before annotation, ORF prediction was performed on all the assembled transcript sequences using TransGeneScan v1.2.1 software.

After quality control, reads were mapped to the representative genes with 95% identity, and FPKM was evaluated using RSEM (http://deweylab.biostat.wisc.edu/rsem/). BLASTP was employed for taxonomic annotations by aligning nonredundant gene catalogs against the NCBI NR database with an e-value cutoff of 1 e^−5^ since the host genome is not published at the present time. Then, the expression abundance profile at the taxonomic level was constructed using the sum of the corresponding gene expression abundance at the taxonomic level of domain, kingdom, phylum, class, order, family, genus, and species. GO annotation was performed using Blast2go (https://www.blast2go.com/ accessed on 8 January 2024), aligning sequences to the GO database. COG annotation was performed using BLASTP against the eggNOG database V5.0 (evolutionary genealogy of genes: Non-supervised Orthologous Groups, http://eggnog.embl.de/ accessed on 8 January 2019) [56]. KEGG annotation was performed using BLASTP against the KEGG database (Kyoto Encyclopedia of Genes and Genomes, http://www.genome.jp/kegg/ accessed on 1 January 2024) [57]. All the blast e-value cutoffs were 1 e^−5^. dbCAN2 [58] was used to identify carbohydrate-active enzymes (CAZyme) [59] within the metatranscriptomic databases using HHMER3. To delineate functional members of the gut microbial community, we cross-referenced the 16S rRNA gene phylogenetic profile. Statistical analyses were performed using R. 

### 4.5. Environmental Data and Statistical Analysis

The data set on China’s regional surface meteorological elements (1979–2018) was obtained from the website (https://data.tpdc.ac.cn/ accessed on 8 January 2024) [60], including 7 elements: near-surface air temperature (temp), near-surface air pressure (pres), near-surface air specific humidity (shum), near-surface wind speed (wind), surface downward shortwave radiation (srad), surface downward longwave radiation (lrad), and precipitation rate (prec). The data were in NETCDF format, with a time resolution of 3 h and a horizontal spatial resolution of 0.1°. Then, we used the FTP client tool to download the data and opened it with Panoply V.5.2.2 software (https://www.giss.nasa.gov/tools/panoply/ accessed on 31 January 2024) and obtained the corresponding meteorological data according to the sample collection time and sites.

Variance inflation factor (VIF) analysis was measured to judge the collinearity between different environmental factors, VIF > 10 was removed, and the screened environmental factors were used for subsequent analysis. Consequently, canonical correlation analysis (CCA) was selected, and then variance partitioning analysis (VPA) was used to quantitatively evaluate the individual and joint interpretations of environmental factor variables on microbial community differences, which was conducted in R package “vegan”. 

### 4.6. Estimation of Ecological Processes and Source Tracking of the Larval Gut Microbiome

We analyzed the bacterial and fungal networks of the coexistence and separate existence sites separately, as well as the network associations between them. This analysis step generated clusters based on the genera shared between the samples, i.e., the more genera shared, the closer the relationship between the samples. The number of nodes and edges, network diameter, average path length, degree, closeness centrality, and betweenness centrality were computed using Networkx and visualized using Cytoscape v3.0.1 [52]. In addition, we used Circos plots to reflect the proportional composition of the dominant genera in the different samples using the Circos-0.67-7 (http://circos.ca/ accessed on 16 March 2015) [61].

A source model of microbiome (SMM), the conceptual model of the plant microbiome source, was constructed based on possible sources of microbial populations and interactions among woodwasp gut and frass microbial communities. Specifically, the Source Tracker v1.0 software (https://github.com/danknights/sourcetracker accessed on 18 September 2016) [62] program and its associated Bayesian algorithm were used to predict the proportion of sink samples from each source based on the community structures of source and sink samples. The female gut, male gut, and frass microbiome community data from the coexistence and separate existence sites served as separate sources, and the larval gut data were used as a sink for each calculation. Of these, the male and female gut data are not shown in detail in this paper.

The estimation of ecological processes was performed according to Stegen et al. [63] and Li et al. [64] (Figure S11). First, both βNTI and RC were calculated using null models (1000 randomizations), with the consideration of OTU abundances (log-transformed) [32]. We then incorporated βNTI and RC to estimate the relative strength of homogeneous selection (βNTI < −2), heterogeneous selection (βNTI > 2), homogeneous dispersal (RC_bary_ < −0.95 and |βNTI| ≤ 2), dispersal limitation (RC_bary_ > 0.95 and |βNTI| ≤ 2), and undominated (also refers to as drift process; |RC| ≤ 0.95 and |βNTI| ≤ 2) in governing the composition of the gut microbiota.

## 5. Conclusions

Our study employed high-throughput metatranscriptomic sequencing to analyze the gut and frass of *S. noctilio* larvae from four sites, involving only *S. noctilio* and both *Sirex* species. We explored the impact of environmental factors, biological interactions, and ecological processes on the assembly of the *S. noctilio* gut microbial community. Amplicon sequencing of two Sirex species, compared to *S. nitobei*, revealed distinct bacterial and fungal composition and functional predictions, and future studies should include more samples for comprehensive comparative analysis. 

*Sirex noctilio* larvae at coexistence sites recruited more microbes from surrounding environmental microbiota than through vertical transmission from adults. Additionally, stochastic processes such as drift and dispersal significantly influenced the *S. noctilio* larval gut microbiome assembly. Moreover, temperature and precipitation positively correlate with most of the highly abundant bacterial and fungal genera.

**Figure 8:**
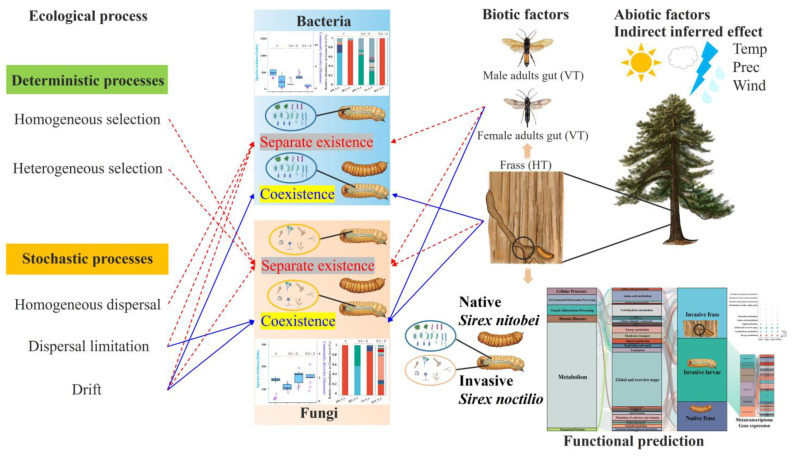
An illustration of the sources and assembly of *Sirex noctilio* gut microbiome in different sites. After the invasion of *S. noctilio* into China, the interaction between abiotic and biotic factors directly or indirectly affects the assembly of their larval gut microbiome, as well as ecological processes. Functional prediction based on amplifier sequencing and metatranscriptome gene expression for larval gut microbiome.

In summary, the *S. noctilio* larval gut microbiome displays a complex interplay of regional and host specificity, environmental factors, and ecological processes, particularly evident after coexistence with native species (Figure 8). Functional predictions and metatranscriptomic analyses from both coexistence and separate existence sites reveal that the majority of metabolic pathways are enriched in energy, carbohydrate, lipid, and amino acid metabolism. Together, these findings offer new insights into the coevolutionary mechanisms between insect hosts and their gut microbiomes and how these microbiomes aid invasive species in successfully establishing and stabilizing in new environments. Future research should delve deeper into key taxonomic groups with metagenomic and metatranscriptomic techniques alongside functional validation of cultivable strains.

## Figures and Tables

**Table 1:** The CAZy family of *Sirex noctilio* gut revealed by the metatranscriptome.

CAZy	Count of Family	Count of Gene	Major Family
GHs	28	99	GH16, GH18, GH20, GH47, GH13, GH38, GH31, GH109, GH15, GH22
GTs	46	173	GT31, GT27, GT2, GT92, GT49, GT8, GT10, GT7, GT1,GT22, GT4
AAs	4	13	AA3, AA4, AA1, AA7
CBMs	19	127	CBM14, CBM32, CBM13, CBM21, CBM48, CBM50, CBM37, CBM63
CEs	7	46	CE10, CE1, CE4, CE14, CE11, CE5, CE9
PLs	2	2	PL1, PL22
	106	460	

**Table 2:** Sample collection information.

Location	Geographic Coordinates	Developmental Stage		Collection Year/Month
		Larva	Frass	
Duerbert Mongolian Autonomous County, Daqing City, Heilongjiang Province (DM)	46.88° N 124.46° E	50	50	May 2018
Hegang City, Heilongjiang Province (HG)	47.12° N 130.17° E	40	40	May 2016
Jinbaotun Town, Tongliao City, Inner Mongolia Autonomous Region (JBT)	43.07° N 123.28^°^ E	35	35	May 2018
Yushu City, Jilin Province (YS)	44.86° N 126.87° E	60	35	May 2018
Zhanggutai Town, Zhangwu County, Fuxin City, Liaoning Province (ZGT)	42.64° N 122.50° E	30	/	May 2018

## Data Availability

All the raw Illumina sequencing data for the Amplicon and metatransgenomic datasets can be found in the NCBI SRA database under GenBank accession numbers PRJNA1046534 and SRP474943.

## Supplementary Materials

The following supporting information can be downloaded at: https://www.mdpi.com/article/10.3390/ijms25052526/s1.
