# Supplementary material for: Functional and Compositional Changes in Sirex noctilio Gut Microbiome in Different Habitats: Unraveling the Complexity of Invasive Adaptation
Source: Int J Mol Sci. 2024 Feb 21;25(5):2526. doi: 10.3390/ijms25052526 (PMC10931295; doi:10.3390/ijms25052526)
Supplement: Supplementary file 1 [file ijms-25-02526-s001.zip › 240203-Suppl.pdf]

Supplementary Material

1 Supplementary Fig.s and Tables

1.1 Supplementary Tables

Supplementary Table S1 Taxonomic information of bacterial and fungal communities in different groups

	Sample	Reads	OTUs	Mean length	Number of different taxonomic categories					
					Phylum	Class	Order	Family	Genus	Species
Bacteria	DM_S_L1	108010	585	426	14	22	48	72	116	151
	DM_S_L2	91406	497	426	13	21	48	69	104	142
	DM_S_L3	79950	427	427	13	19	44	61	94	118
	DM_S_L4	28524	180	425	10	16	35	49	65	79
	DM_S_L5	90660	598	394	23	43	90	125	191	244
	DM_S_FR1	69932	394	409	20	34	78	126	209	283
	DM_S_FR2	68187	495	409	20	34	83	134	249	346
	DM_S_FR3	61243	251	408	13	20	57	86	142	186
	DM_S_FR4	57639	460	411	18	29	74	128	234	313
	DM_S_FR5	64232	450	417	21	34	73	116	213	304
	HG_S_L1	59478	525	429	19	29	84	146	283	404
	HG_S_L2	52567	355	432	13	23	63	115	207	286
	HG_S_L3	46301	83	425	9	12	28	44	63	75
	HG_S_L4	52616	34	425	8	10	21	25	26	31
	HG_S_FR1	32933	160	443	13	17	39	61	102	124
	HG_S_FR2	95822	855	435	22	37	90	155	279	432
	HG_S_FR3	96050	739	437	20	44	98	158	263	401
	HG_S_FR4	51497	536	415	22	41	90	154	272	357
	JBT_S_L1	63951	152	415	13	20	43	70	102	120
	JBT_S_L2	69439	161	410	13	22	53	75	105	126
	JBT_S_L3	66021	147	411	17	20	47	71	93	108
	JBT_S_FR1	42092	750	427	33	79	18	265	419	612
	JBT_S_FR2	9315	619	415	26	66	14	222	364	500
	JBT_S_FR3	33410	96	429	15	24	46	64	79	83
	YS_S_L1	63332	338	411	20	31	71	114	200	257
	YS_S_L2	60982	438	412	20	39	96	156	258	346
	YS_S_L3	49638	376	410	23	43	92	141	229	312
	YS_S_L4	58038	315	409	18	33	84	142	219	271
	YS_S_FR1	62769	196	407	12	18	46	75	116	148
	YS_S_FR2	72564	210	408	12	18	49	80	122	151
	YS_S_FR3	64556	248	408	16	20	51	80	133	175
	ZGT_X_L1	51741	91	404	12	16	31	42	59	69
	ZGT_X_L2	61523	155	404	13	22	45	67	93	110
	ZGT_X_L3	60083	81	404	8	11	26	43	57	64
	ZGT_X_L4	43579	137	404	10	17	42	62	86	103
	ZGT_X_L5	51668	137	405	14	19	42	62	88	104
	ALL		4912	416	44	121	292	503	1154	2079
Fungi	DM_S_L1	67308	137	325	3	12	18	25	28	35
	DM_S_L2	62800	147	330	3	11	20	30	33	37
	DM_S_L3	88724	157	330	2	4	5	6	6	8
	DM_S_L4	81254	127	330	3	8	12	15	15	17
	DM_S_L5	89472	141	330	2	3	4	5	5	7

DM_S_FR1	65923	118	330	2	7	9	10	10	12
DM_S_FR2	65303	98	330	2	3	4	4	4	5
DM_S_FR3	69994	132	330	2	10	17	28	30	39
JBT_S_L1	73372	137	316	2	11	23	42	51	66
JBT_S_L2	54414	91	313	2	11	25	34	40	48
JBT_S_L3	69657	104	313	2	10	22	33	40	51
JBT_S_FR1	123966	116	311	2	2	3	3	5	6
JBT_S_FR2	136722	121	313	2	7	14	17	20	23
JBT_S_FR3	144756	117	313	2	3	4	5	6	7
JBT_S_FR4	65532	51	313	2	2	3	4	5	7
YS_S_L1	64109	165	325	3	14	29	40	49	62
YS_S_L2	73063	177	340	3	16	36	51	61	77
YS_S_L3	69347	91	399	2	11	25	40	46	59
YS_S_FR1	144834	168	320	2	5	5	5	6	9
YS_S_FR2	85954	158	321	2	3	4	4	5	8
YS_S_FR3	125140	118	315	2	4	5	5	6	9
ZGT_X_L1	83838	194	338	5	11	15	17	21	30
ZGT_X_L2	108128	165	325	3	9	25	36	41	55
ZGT_X_L3	149940	151	316	2	8	10	15	16	21
ZGT_X_L4	145446	151	312	2	8	17	21	24	31
ZGT_X_L5	72476	120	339	2	7	10	15	19	26
ALL		1432	326	6	27	67	131	188	255

Supplementary Table S2 Summary of observed bacterial and fungal OTUs, Shannon, Simpson, Chao, ACE and Coverage

Sample	Bacteria					Sample	Fungi				
	Shannon	Simpson	ACE	Chao	Coverage		Shannon	Simpson	ACE	Chao	Coverage
DM_S_L1	2.42	0.27	587.00	587.00	1.00	DM_S_L1	0.59	0.78	139.00	139.00	1.00
DM_S_L2	2.50	0.21	515.00	515.00	1.00	DM_S_L2	0.31	0.92	150.00	150.00	1.00
DM_S_L3	2.41	0.24	433.00	433.00	1.00	DM_S_L3	0.18	0.96	157.00	157.00	1.00
DM_S_L4	2.53	0.22	212.51	210.67	1.00	DM_S_L4	0.14	0.97	127.00	127.00	1.00
DM_S_L5	3.30	0.17	605.00	605.00	1.00	DM_S_L5	0.19	0.95	141.00	141.00	1.00
DM_S_FR1	2.59	0.29	480.10	492.88	1.00	DM_S_FR1	0.26	0.93	221.63	215.60	1.00
DM_S_FR2	2.97	0.24	586.29	598.24	1.00	DM_S_FR2	0.25	0.94	122.05	120.23	1.00
DM_S_FR3	1.69	0.43	324.22	329.11	1.00	DM_S_FR3	0.31	0.92	182.18	186.65	1.00
DM_S_FR4	3.82	0.09	532.94	553.12	1.00						
DM_S_FR5	3.56	0.09	538.75	571.20	1.00						
HG_S_L1	1.86	0.56	578.62	598.55	1.00						
HG_S_L2	2.06	0.32	475.98	485.35	1.00						
HG_S_L3	0.17	0.96	252.80	182.00	1.00						
HG_S_L4	0.08	0.98	227.07	80.20	1.00						
HG_S_FR1	2.81	0.09	369.80	271.24	1.00						
HG_S_FR2	4.88	0.03	859.00	859.00	1.00						
HG_S_FR3	5.07	0.02	792.00	792.00	1.00						
HG_S_FR4	4.38	0.05	606.12	621.82	1.00						
JBT_S_L1	1.84	0.26	282.19	243.06	1.00	JBT_S_L1	1.46	0.34	167.85	179.40	1.00
JBT_S_L2	1.85	0.33	323.86	275.40	1.00	JBT_S_L2	0.72	0.74	108.98	122.88	1.00
JBT_S_L3	2.10	0.25	232.89	223.27	1.00	JBT_S_L3	1.08	0.44	128.28	130.43	1.00
JBT_S_FR1	1.89	0.52	804.99	796.47	1.00	JBT_S_FR1	1.03	0.44	116.00	116.00	1.00
JBT_S_FR2	5.19	0.03	645.05	651.02	0.99	JBT_S_FR2	0.45	0.82	124.00	124.00	1.00
JBT_S_FR3	0.56	0.72	935.23	288.15	1.00	JBT_S_FR3	1.07	0.42	117.00	117.00	1.00
						JBT_S_FR4	0.45	0.84	82.50	96.50	1.00

YS_S_L1	3.26	0.11	455.83	467.75	1.00	YS_S_L1	1.22	0.50	202.88	199.12	1.00
YS_S_L2	3.04	0.18	557.76	565.06	1.00	YS_S_L2	2.76	0.12	273.14	246.13	1.00
YS_S_L3	2.25	0.30	447.94	456.46	1.00	YS_S_L3	1.11	0.46	101.52	110.60	1.00
YS_S_L4	1.98	0.33	453.45	432.68	1.00						
YS_S_FR1	1.42	0.48	283.59	303.81	1.00	YS_S_FR1	1.10	0.43	168.00	168.00	1.00
YS_S_FR2	1.49	0.46	285.16	330.55	1.00	YS_S_FR2	1.08	0.44	158.00	158.00	1.00
YS_S_FR3	2.01	0.35	331.66	335.65	1.00	YS_S_FR3	0.85	0.64	118.00	118.00	1.00
ZGT_X_L1	0.19	0.94	277.09	162.30	1.00	ZGT_X_L1	2.13	0.25	209.00	209.00	1.00
ZGT_X_L2	0.24	0.93	349.74	271.37	1.00	ZGT_X_L2	3.29	0.08	210.00	210.00	1.00
ZGT_X_L3	0.21	0.93	142.08	126.35	1.00	ZGT_X_L3	0.85	0.71	163.00	163.00	1.00
ZGT_X_L4	0.25	0.92	290.79	221.44	1.00	ZGT_X_L4	1.16	0.43	158.00	158.00	1.00
ZGT_X_L5	0.35	0.89	331.69	217.47	1.00	ZGT_X_L5	1.62	0.48	137.00	137.00	1.00

Supplementary Table S3 Statistical test for species richness and community diversity among different woodwasp species (one-way ANOVA, LSD post-hoc test).

A) Bacteria

sobs	Df	Sum Sq	Mean Sq	F value	Pr(>F)	Sig.
group	8	945975	118247	4.544	0.00136	**
Residuals	27	702609	26023			

Shannon	Df	Sum Sq	Mean Sq	F value	Pr(>F)	Sig.
group	8	80.51	10.064	16.48	1.50E-08	***
Residuals	27	16.48	0.611			

B) Fungi

sobs	Df	Sum Sq	Mean Sq	F value	Pr(>F)	Sig.
group	6	10533	1755.5	2.349	0.0724	.
Residuals	19	14200	747.4			

Shannon	Df	Sum Sq	Mean Sq	F value	Pr(>F)	Sig.
group	6	8.625	1.4374	4.805	0.00386	**
Residuals	19	5.684	0.2991			

Signif. codes: 0 ‘***’ 0.001 ‘**’ 0.01 ‘*’ 0.05 ‘.’ 0.1

Supplementary Table S4 The major bacterial microorganisms (BM) and their relative abundance (RA) of five taxonomic levels of *Sirex noctilio* and *Sirex nitobei* gut and frass (for median)

Sample	Phylum		Class		Order		Family	
	BM	RA(%)	BM	RA(%)	BM	RA(%)	BM	RA(%)
DM_S_L	Proteobacteria	92.7	Gammaproteobacteria	88.7	Pseudomonadales	66.1	Pseudomonadaceae	66.9
	Actinobacteria	5.3	Actinobacteria	5.3	Burkholderiales	22.3	Burkholderiaceae	17.6
			Alphaproteobacteria	4.1	Corynebacteriales	4.7	Nocardiaceae	4.8
					Rhizobiales	3.4	Comamonadaceae	2.3
							Beijerinckiaceae	2.1
							Oxalobacteraceae	1.6
DM_S_FR	Proteobacteria	65.8	Alphaproteobacteria	38.5	Pseudomonadales	16.6	Acetobacteraceae	10.7
	Actinobacteria	12.8	Gammaproteobacteria	28.9	Rhizobiales	13.9	Pseudomonadaceae	10.2
	Acidobacteria	8.7	Actinobacteria	10.1	Burkholderiales	11.2	Beijerinckiaceae	10.1
	Bacteroidetes	6.3	Acidobacteriia	8.7	Acetobacterales	9.6	Acidobacteriaceae	8.0
	Firmicutes	2.7	Bacteroidia	6.6	Acidobacteriales	7.4	Burkholderiaceae	6.5
			Bacilli	1.5	Xanthomonadales	4.7	Moraxellaceae	6.4
HG_S_L	Proteobacteria	95.8	Alphaproteobacteria	92.2	Rickettsiales	93.3	Anaplasmataceae	94.0
	Actinobacteria	2.2	Gammaproteobacteria	3.8			others	1.5
	Firmicutes	1.7	Actinobacteria	2.2				
			Bacilli	1.4				
HG_S_FR	Proteobacteria	62.7	Gammaproteobacteria	42.5	Burkholderiales	11.1	Sphingomonadaceae	11.6
	Firmicutes	11.9	Alphaproteobacteria	20.0	Xanthomonadales	11.0	Burkholderiaceae	8.4
	Actinobacteria	11.1	Actinobacteria	10.6	Sphingomonadales	10.7	Rhodanobacteraceae	7.8
	Bacteroidetes	7.8	Bacteroidia	8.1	Pseudomonadales	7.7	Pseudomonadaceae	7.3
	Acidobacteria	4.1	Clostridia	6.4	Enterobacterales	7.5	Acidobacteriaceae	4.8
			Bacilli	5.5	Rhizobiales	6.9	others	14.0
JBT_S_L	Proteobacteria	46.2	Bacteroidia	54.3	Chitinophagales	57.0	Chitinophagaceae	58.4
	Bacteroidetes	45.4	Gammaproteobacteria	19.5	Enterobacterales	7.4	Acetobacteraceae	6.4

	Actinobacteria	2.2	Alphaproteobacteria	15.8	Acetobacterales	6.3	Enterobacteriaceae	5.5
	Acidobacteria	2.0	Actinobacteria	2.6	Xanthomonadales	6.0	Rhodanobacteraceae	4.9
	Firmicutes	1.7	Acidobacteriia	2.4	Rhizobiales	5.6	Beijerinckiaceae	2.8
JBT_S_FR	Proteobacteria	89.7	Gammaproteobacteria	87.8	Pseudomonadales	46.1	Pseudomonadaceae	49.3
	Actinobacteria	3.5	Actinobacteria	2.7	Burkholderiales	9.3	Rhodanobacteraceae	7.7
	Bacteroidetes	1.8	Alphaproteobacteria	2.4	Xanthomonadales	7.6	Sphingomonadaceae	5.6
	Chloroflexi	1.5	Bacteroidia	1.7	Corynebacteriales	5.4	Nocardiaceae	5.5
	Firmicutes	1.3	others	3.8	Sphingomonadales	5.2	Burkholderiaceae	3.9
					Chitinophagales	2.0	others	16.5
YS_S_L	Proteobacteria	45.7	Alphaproteobacteria	29.1	Chitinophagales	22.8	Chitinophagaceae	24.1
	Bacteroidetes	24.0	Bacteroidia	24.2	Burkholderiales	9.7	Deinococcaceae	8.4
	Actinobacteria	10.6	Gammaproteobacteria	17.0	Deinococcales	7.9	Acetobacteraceae	7.9
	Firmicutes	8.8	Actinobacteria	9.7	Acetobacterales	7.5	Microbacteriaceae	6.3
	Deincoccota	7.5	Bacilli	7.6	Micrococcales	6.8	Oxalobacteraceae	5.5
			Deinococci	7.5	Rhizobiales	5.7	others	7.3
YS_S_FR	Proteobacteria	67.9	Gammaproteobacteria	40.2	Pseudomonadales	35.2	Pseudomonadaceae	23.1
	Actinobacteria	14.4	Alphaproteobacteria	27.5	Rhizobiales	15.5	Moraxellaceae	12.2
	Bacteroidetes	7.8	Actinobacteria	14.1	Corynebacteriales	5.7	Beijerinckiaceae	10.9
	Firmicutes	5.0	Bacteroidia	7.8	Chitinophagales	5.7	Chitinophagaceae	5.9
	Acidobacteria	2.1	Bacilli	4.2	Sphingomonadales	4.6	Sphingomonadaceae	4.8
			Acidobacteriia	2.2	Micrococcales	4.4	others	7.5
ZGT_X_L	Proteobacteria	99.6	Alphaproteobacteria	98.8	Rickettsiales	98.9	Anaplasmataceae	99.0
			Gammaproteobacteria	0.7				

Supplementary Table S5 The major fungal microorganisms (FM) and their relative abundance (RA) of five taxonomic levels of *Sirex noctilio* and *Sirex nitobei* gut and frass（for median）

Sample	Phylum		Class		Order		Family		Genus	
	FM	RA(%)	FM	RA(%)	FM	RA(%)	FM	RA(%)	FM	RA(%)
DM_S_L	Basidiomycota	99.9	Agaricomycetes	99.9	Russulales	99.9	Stereaceae	99.9	*Amylostereum*	99.9
DM_S_FR	Basidiomycota	99.9	Agaricomycetes	99.9	Russulales	99.9	Stereaceae	99.9	*Amylostereum*	99.9
JBT_S_L	Ascomycota	99.8	Sordariomycetes	99.1	Ophiostomatales	58.1	Ophiostomataceae	58.2	*Ophiostoma*	58.2
					Hypocreales	41.1	Hypocreaceae	41.1	*Trichoderma*	41.1
JBT_S_FR	Ascomycota	99.9	Sordariomycetes	99.9	Ophiostomatales	70.8	Ophiostomataceae	97.6	*Ophiostoma*	97.5
					Hypocreales	29.1	Hypocreaceae	2.1	*Trichoderma*	2.1
YS_S_L	Ascomycota	67.3	Sordariomycetes	62.3	Ophiostomatales	47.9	Ophiostomataceae	49.8	F_Ophiostomataceae	49.3
	Basidiomycota	32.7	Agaricomycetes	26.0	Russulales	33.2	Stereaceae	34.5	*Amylostereum*	34.7
			Dothideomycetes	7.0	Botryosphaeriales	6.7	Botryosphaeriaceae	7.0	*Diplodia*	7.0
			Tremellomycetes	1.4	Pleosporales	4.3	Tremellaceae	1.2	*Tremella*	1.2
			Eurotiomycetes	1.3	Tremellales	1.2	Cladosporiaceae	1.0	*Cladosporium*	1.0
							others	2.9	others	2.6
YS_S_FR	Ascomycota	62.5	Sordariomycetes	61.2	Ophiostomatales	61.1	Ophiostomataceae	61.1	F_Ophiostomataceae	60.5
	Basidiomycota	37.5	Agaricomycetes	37.5	Russulales	37.4	Stereaceae	37.4	*Amylostereum*	37.5
			Dothideomycetes	1.4	Botryosphaeriales	1.4	Botryosphaeriaceae	1.4	*Diplodia*	1.4
ZGT_X_L	Ascomycota	90.5	Sordariomycetes	57.6	P_Ascomycota	32.3	P_Ascomycota	38.3	P_Ascomycota	47.9
	Basidiomycota	9.5	P_Ascomycota	18.5	Hypocreales	22.8	Ophiostomataceae	18.0	*Amylostereum*	21.4
			Agaricomycetes	14.3	Russulales	15.4	Stereaceae	17.1	F_Ophiostomataceae	6.8
			Dothideomycetes	4.3	Ophiostomatales	15.2	Nectriaceae	9.3	*Malassezia*	4.5
			Eurotiomycetes	3.6	Eurotiales	5.4	Aspergillaceae	6.4	*Penicillium*	4.1

Supplementary Table S6 The R value and P value of the correlation coefficient between *Sirex noctilio* larval gut and frass bacterial genera in the top 50 relative abundance

(see TABLE S6 BACTERIA-L-FR.XLSX)

Supplementary Table S7 The R value and P value of the correlation coefficient between *Sirex noctilio* larval gut and frass fungal genera in the top 50 relative abundance

(see TABLE S7 FUNGI-L-FR.XLSX)

Supplementary Table S8 Results of permutational multivariate analysis of variance (PERMANOVA)

Bacterial community			Fungal community		
**climate factors**	**R^2**	***p*-Value**	**climate factors**	**R^2**	***p*-Value**
wind	0.6627	0.008	wind	0.9987	0.008
temp	0.9395	0.001	temp	0.8235	0.006
prec	0.9559	0.001			

Supplementary Table S9 The shared and exclusive bacterial microorganisms of *Sirex noctilio* and *Sirex nitobei* larval gut and frass at genus level

(see TABLE S9.XLSX)

Supplementary Table S10 The shared and exclusive fungal microorganisms of *Sirex noctilio* and *Sirex nitobei* larval gut and frass at genus level

(see TABLE S10.XLSX)

Supplementary Table S11 Functional prediction of 16S rRNA, ITS2 and metatranscriptome sequences

(see TABLE S11.XLSX)

Supplementary Table S12 KEGG pathway and KEGG level 2 pathway for Figure 7C, 7D

KEGG pathway	KEGG level 2 pathway
Cellular Processes	Folding, sorting and degradation
	Aging
	Amino acid metabolism
	Biosynthesis of other secondary metabolites
	Cancers: Overview
	Cancers: Specific types
Environmental Information Processing	Carbohydrate metabolism
	Cardiovascular diseases
	Cell growth and death
	Cell motility
	Cellular community - eukaryotes
	Cellular community - prokaryotes
	Transport and catabolism
	Circulatory system
	Development and regeneration
Genetic Information Processing	Digestive system
	Drug resistance: Antineoplastic
	Endocrine and metabolic diseases
	Endocrine system
	Energy metabolism

Human Diseases	Membrane transport
	Signal transduction
	Signaling molecules and interaction
	Environmental adaptation
	Excretory system
	Replication and repair
	Transcription
Metabolism	Translation
	Global and overview maps
	Glycan biosynthesis and metabolism
	Immune diseases
	Infectious diseases: Bacterial
	Infectious diseases: Parasitic
	Infectious diseases: Viral
	Neurodegenerative diseases
	Substance dependence
	Immune system
	Lipid metabolism
	Mtabolism of cofactors and vitamins
	Metabolism of other amino acids
	Metabolism of terpenoids and polyketides
	Nucleotide metabolism
	Xenobiotics biodegradation and metabolism
Organismal Systems	Nervous system
	Sensory system

Supplementary Table S13 Summary of metatranscriptome sequence data obtained from *Sirex noctilio* larval gut

(see TABLE S13.XLSX)

Supplementary Table S14 List of metatranscriptome sequencing genes in *Sirex noctilio* larval gut annotated in the present study.

(see TABLE S14.XLSX)

Supplementary Table S15 NR annotation of *Sirex noctilio* larval gut metatranscriptome sequencing

Major phyla	Number of reads			
	YSL1	YSL2	YSL3	YSL4
Arthropoda	547805.3	572636.2	569332.4	588545.9
Nematoda	7896.536	30011.95	31133.42	18058.13
Proteobacteria	4610.694	1860.102	1775.314	1935.937
Mollusca	313.108	4879.263	1722.561	1465.65
Chordata	993.939	1701.213	1703.066	1385.745
Apicomplexa	1011.517	999.217	1191.222	816.375
Streptophyta	332.255	1247.482	681.838	421.72
Firmicutes	500.091	583.572	812.684	653.258
Cnidaria	343.2	379.319	422.629	280.647
Ascomycota	231.251	324.58	380.55	325.251
Platyhelminthes	165.494	211.615	215.341	195.251
Annelida	160.483	150.633	194.177	127.104
Actinobacteria	153.11	1.688	8.245	0
Glomeromycota	19.4	34.324	36.564	16.802
Cyanobacteria	55.365	5.958	1.621	3.555

Supplementary Table S16 The major bacterial and fungal microorganisms of *Sirex noctilio* larval gut metatranscriptome sequencing

	Major phylum	Count of genes	Major genus
**Bacteria**	Proteobacteria	324	*Rickettsia*<br>*Xanthomonas*<br>*Wolbachia*<br>*Pseudomonas*<br>*Nitrosomonas*<br>*Piscirickettsia*<br>*Anaplasma*<br>*Comamonas*<br>*Bosea*<br>*Xylella*<br>*Pandoraea*

	Firmicutes	78	*Bacillus*<br>*Streptococcus*<br>*Paenibacillus*<br>*Oribacterium*<br>*Pseudoflavonifractor*
	Actinobacteria	37	*Mycobacterium*<br>*Rhodococcus*<br>*Arthrobacter*
	Cyanobacteria	3	*Leptolyngbya*<br>*Crocosphaera*
**Fungi**	Ascomycota	15	*Penicillium*<br>*Aspergillus*<br>*Microsporum*<br>*Trichophyton*<br>*Beauveria*
	Glomeromycota	2	*Rhizophagus*

Supplementary Table S17 Primer sequences used in this study

Primer name	Primer sequence (5'-3')	length
LCO1490	GGTCAACAAATCATAAAGATATTGG	700bp
HCO2198	TAAACTTCAGGGTGACCAAAAAATCA	
16S-338F	ACTCCTACGGGAGGCAGCAG	468bp
16S-806R	GGACTACHVGGGTWTCTAAT	
ITS3F	GCATCGATGAAGAACGCAGC	200~500bp
ITS4R	TCCTCCGCTTATTGATATGC	

1.2 Supplementary Figures

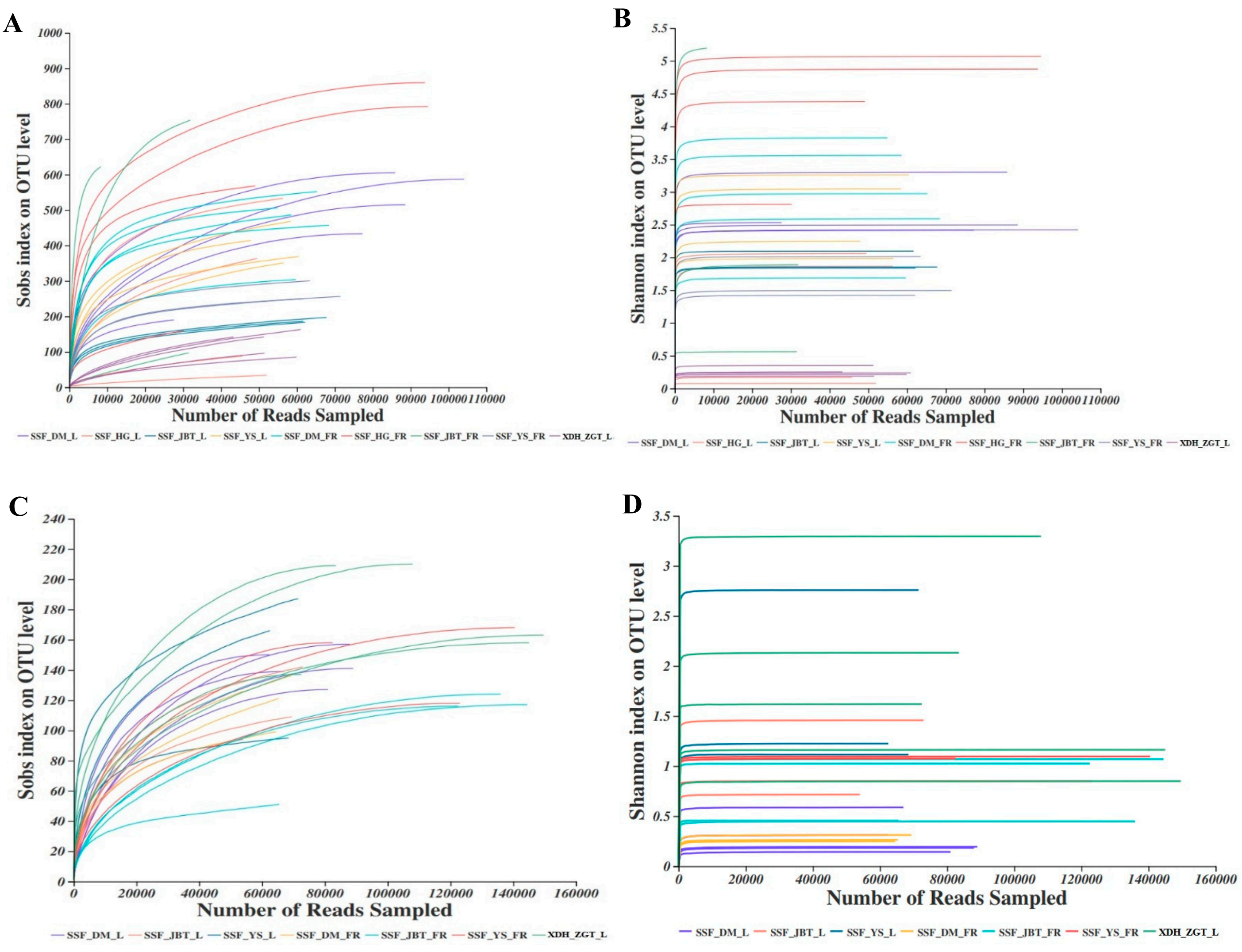

Supplementary Fig. S1 Rarefaction curves of *Sirex noctilio* and *Sirex nitobei* larval gut and frass bacterial and fungal communities at OTU level. (A, C) Sobs index and (B, D) Shannon index. A-B, bacteria, C-D, fungi. SSF, *Sirex noctilio*; XDH, *Sirex nitobei*; L, larva; FR, frass.

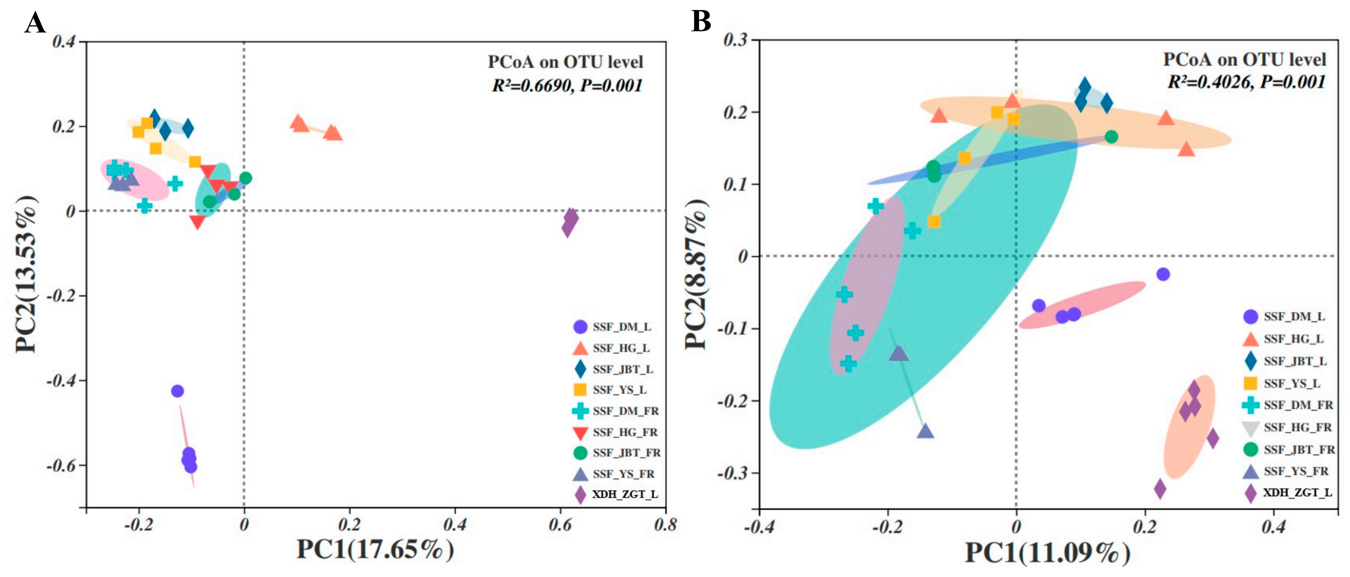

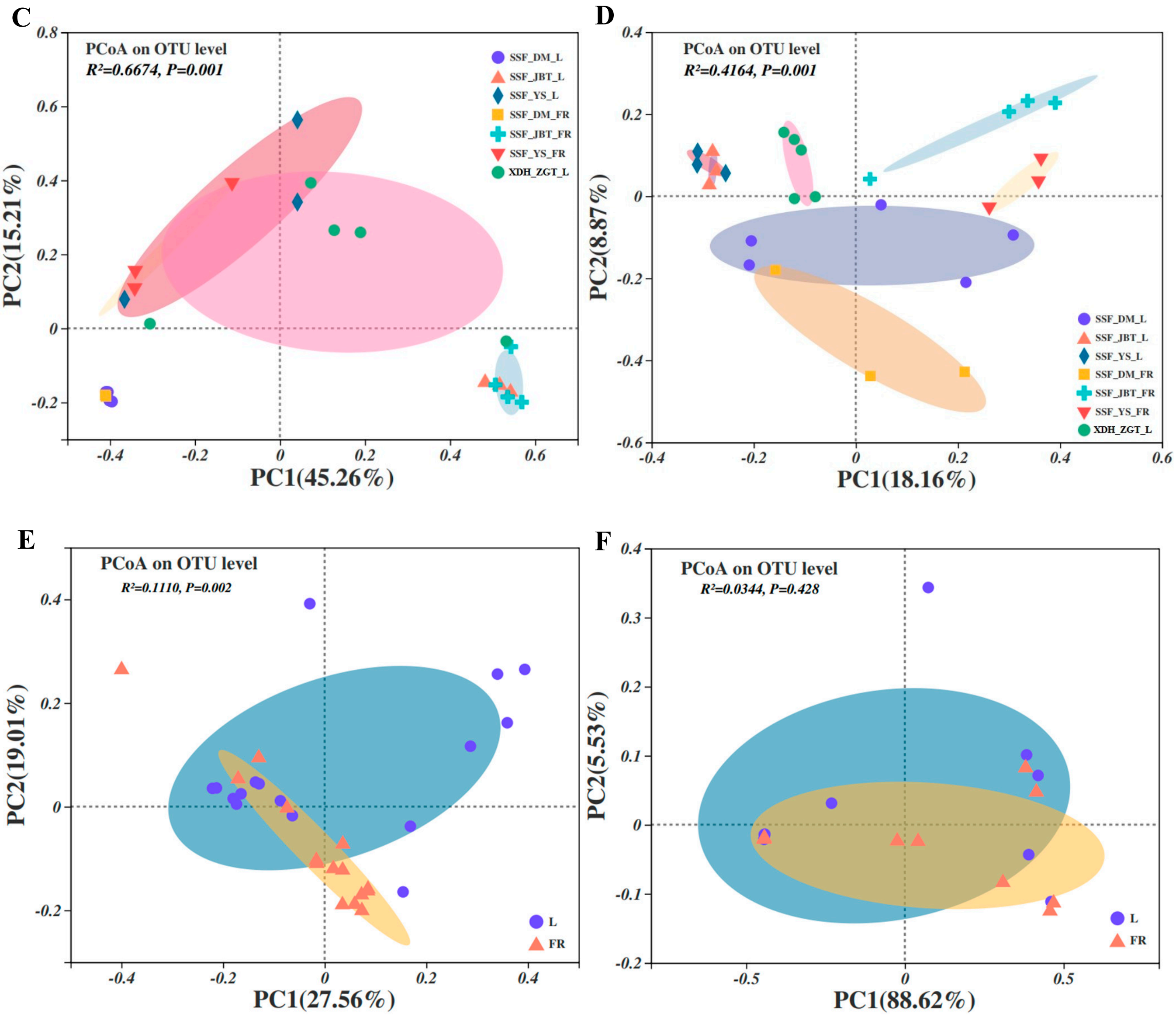

Supplementary Fig. S2 PCoA analysis of *Sirex noctilio* and *Sirex nitobei* larval gut and frass. PCoA plot using (A, C) Bray-Curits, (B, D) unweighted UniFrac, (E, F) weighted UniFrac distance between different groups. PERMANOVA (Adonis) was used for significance analysis, permutation=999. A-B, E, bacteria, C-D, F, fungi; E, F, *Sirex noctilio* larval gut and frass. SSF, *Sirex noctilio*; XDH, *Sirex nitobei*; L, larva; FR, frass.

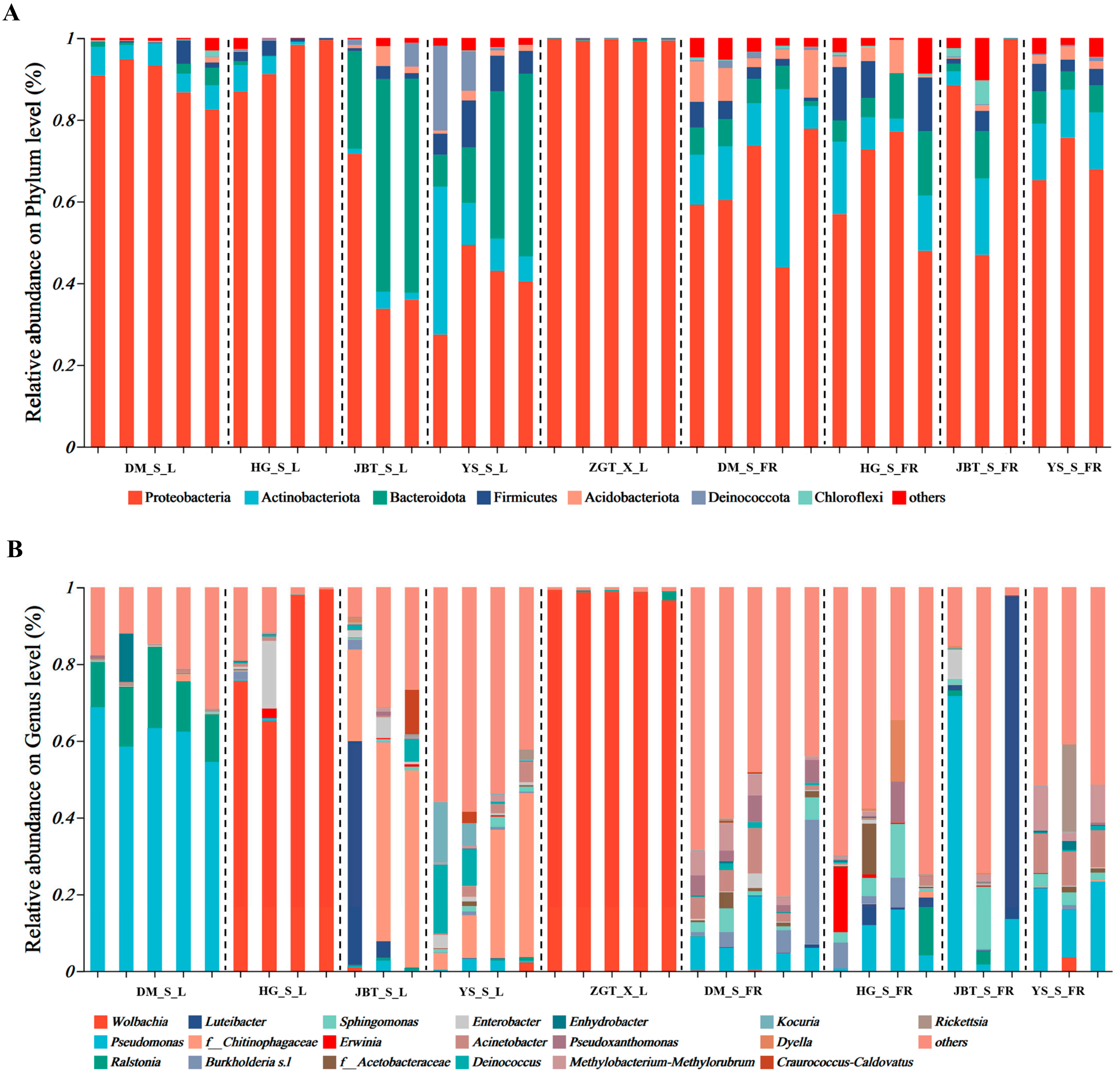

Supplementary Fig. S3 Relative abundance of bacterial communities associated with *Sirex noctilio* and *Sirex nitobei* larval gut and frass. OTUs that were < 5% of average relative abundance in groups are summarized as “others” at the phylum level (A). OTUs that were < 10% of average relative abundance in groups are summarized as “others” at the genus level (B). S, *Sirex noctilio*; X, *Sirex nitobei*; L, larva; FR, frass.

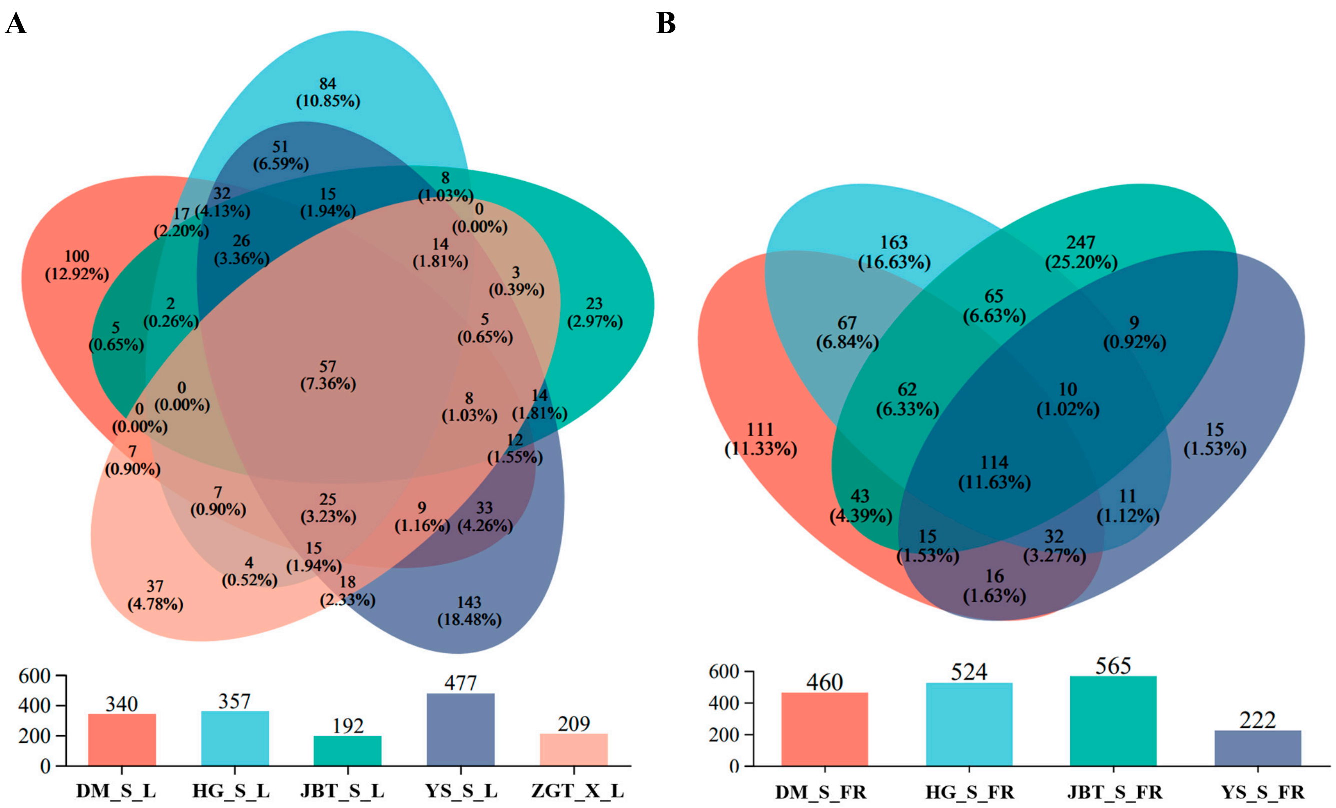

Supplementary Fig. S4 Venn diagrams depicting the overlap of the bacterial community associated with *Sirex noctilio* and *Sirex nitobei* (A) larval gut and (B) frass at the genus level. S, *Sirex noctilio*; X, *Sirex nitobei*; L, larva; FR, frass.

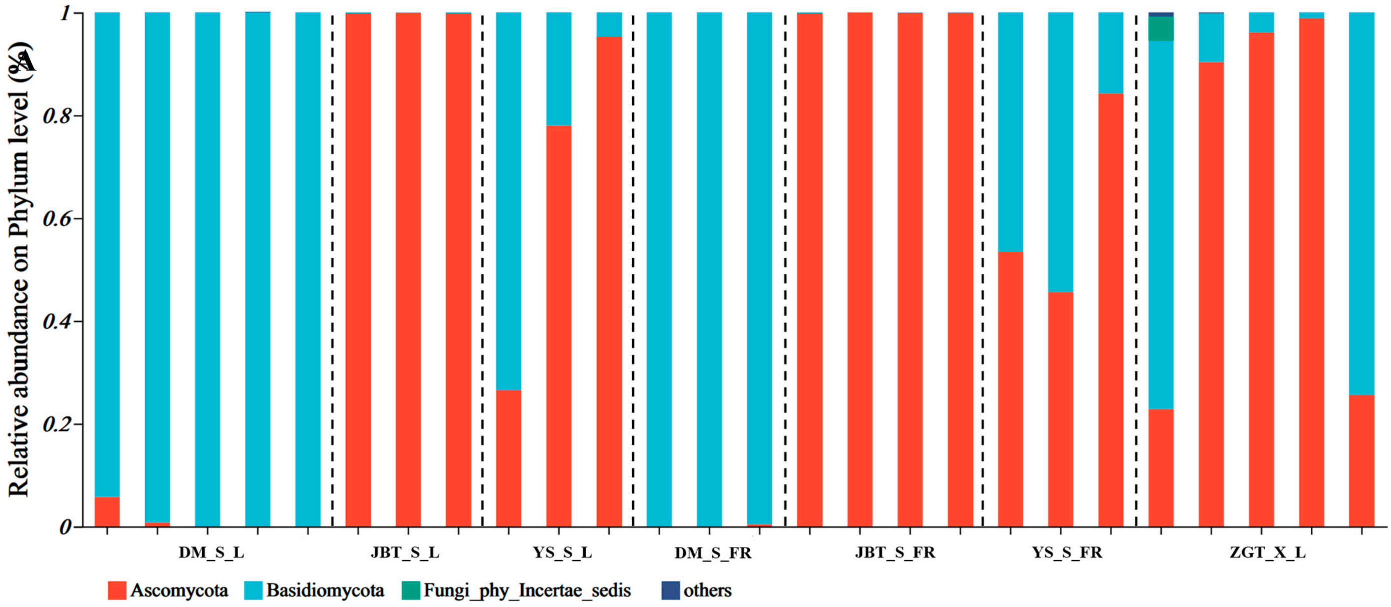

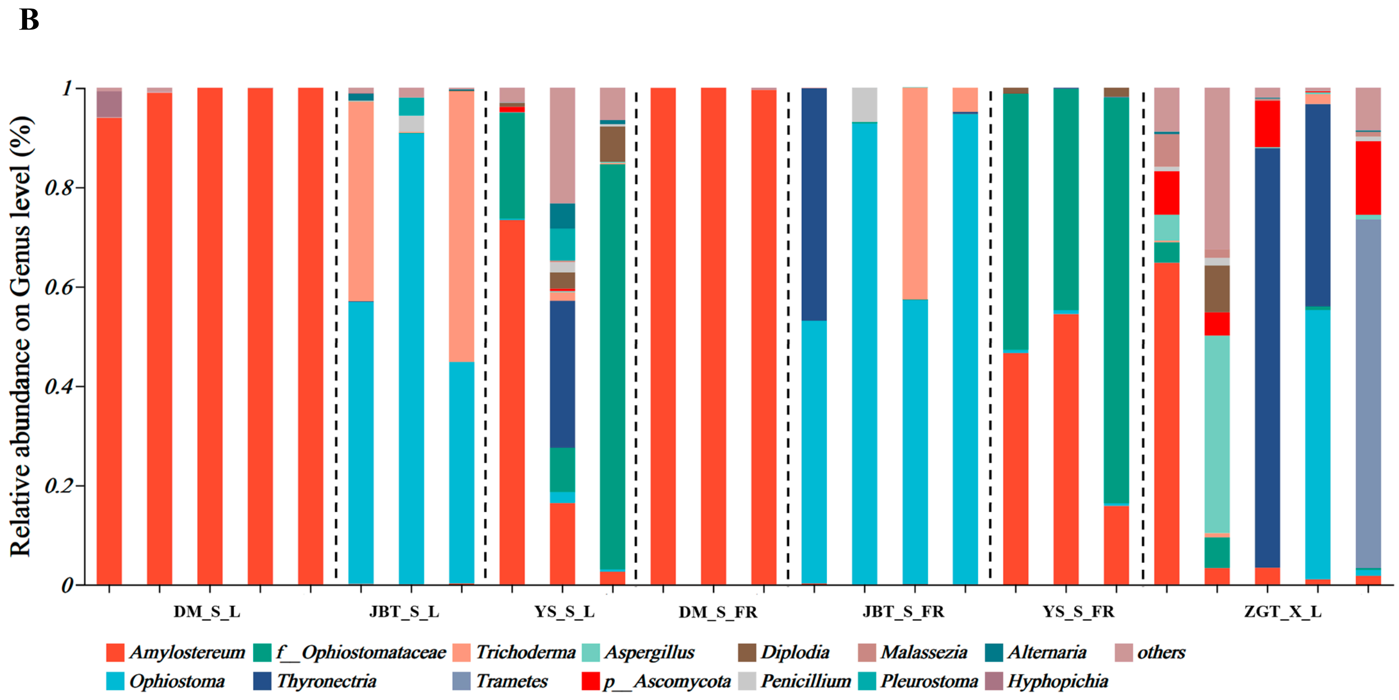

Supplementary Fig. S5 Relative abundance of fungal communities associated with *Sirex noctilio* and *Sirex nitobei* larval gut and frass. OTUs that were < 1% of average relative abundance in groups are summarized as "others" at the phylum level (A). OTUs that were < 5% of average relative abundance in groups are summarized as "others" at the genus level (B). S, *Sirex noctilio*; X, *Sirex nitobei*; L, larva; FR, frass.

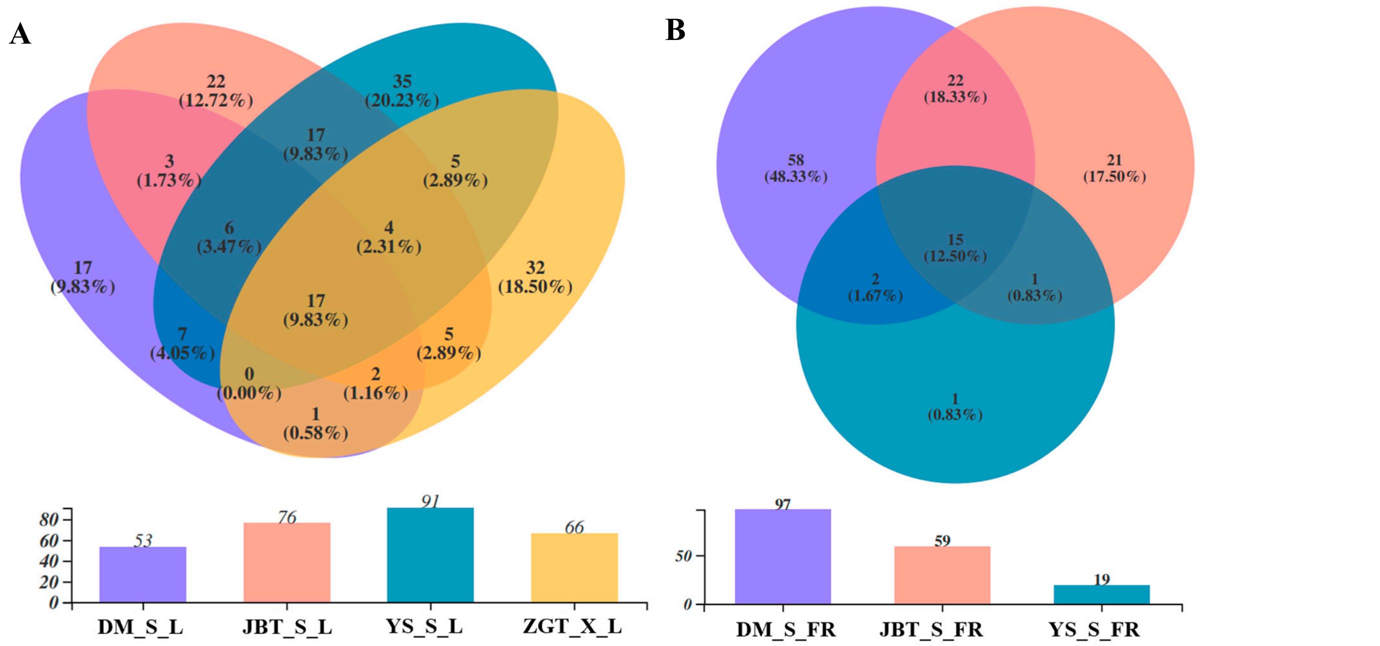

Supplementary Fig. S6 Venn diagrams depicting the overlap of the fungal community associated with *Sirex noctilio* and *Sirex nitobei* (A) larval gut and (B) frass at the genus level. S, *Sirex noctilio*; X, *Sirex nitobei*; L, larva; FR, frass.

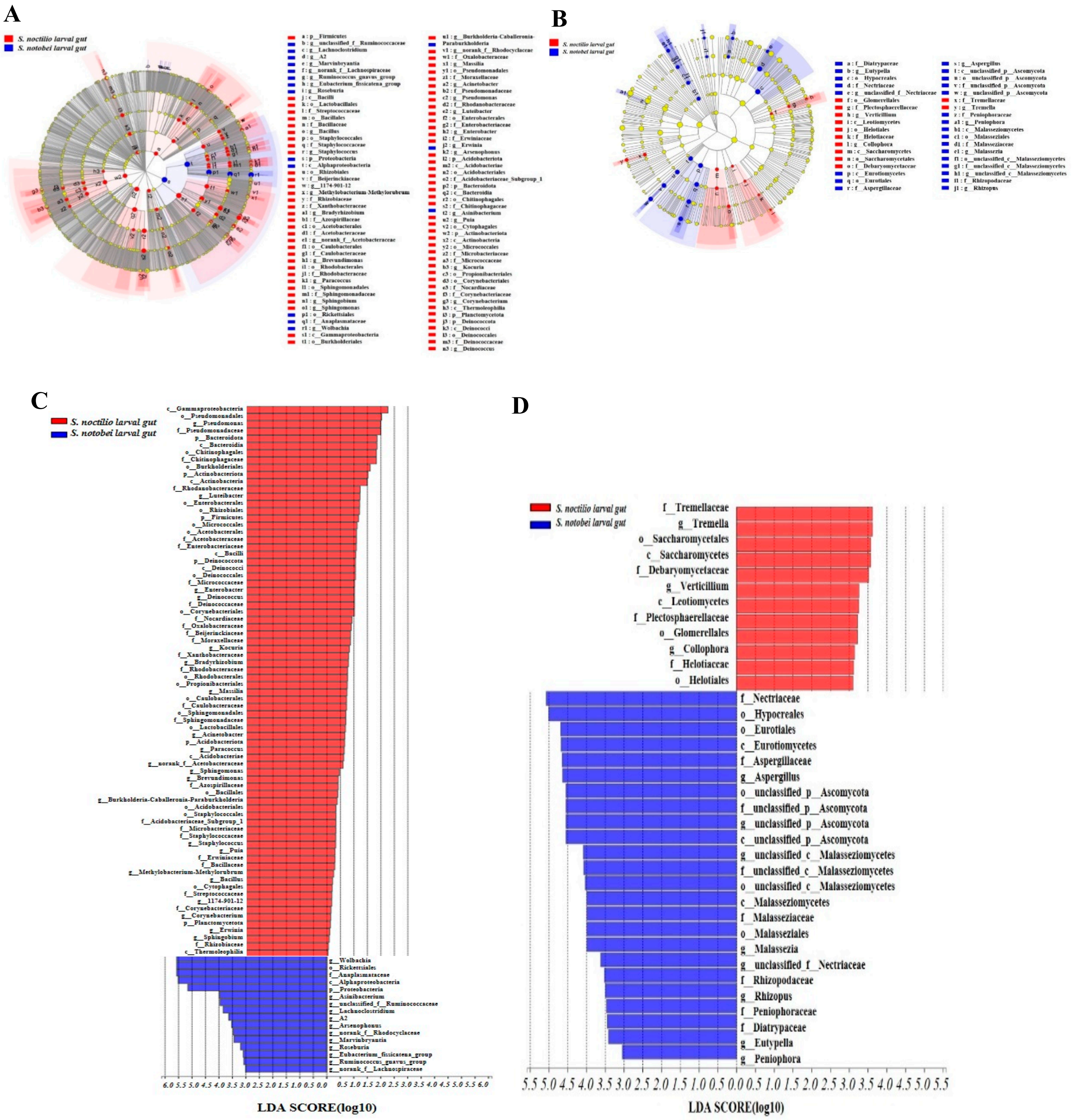

Supplementary Fig. S7 LEfSe (A-B) and linear discriminatory analysis (C-D) showing the different comparison of the two *Sirex* species samples at all levels. The differences in relative abundance of larval gut bacterial (A, C) and fungal (B, D) between *S. noctilio* and *S. nitobei*. LDA score > 3, letters p, c, o, f and g indicate phylum, class, order, family and genus, respectively.

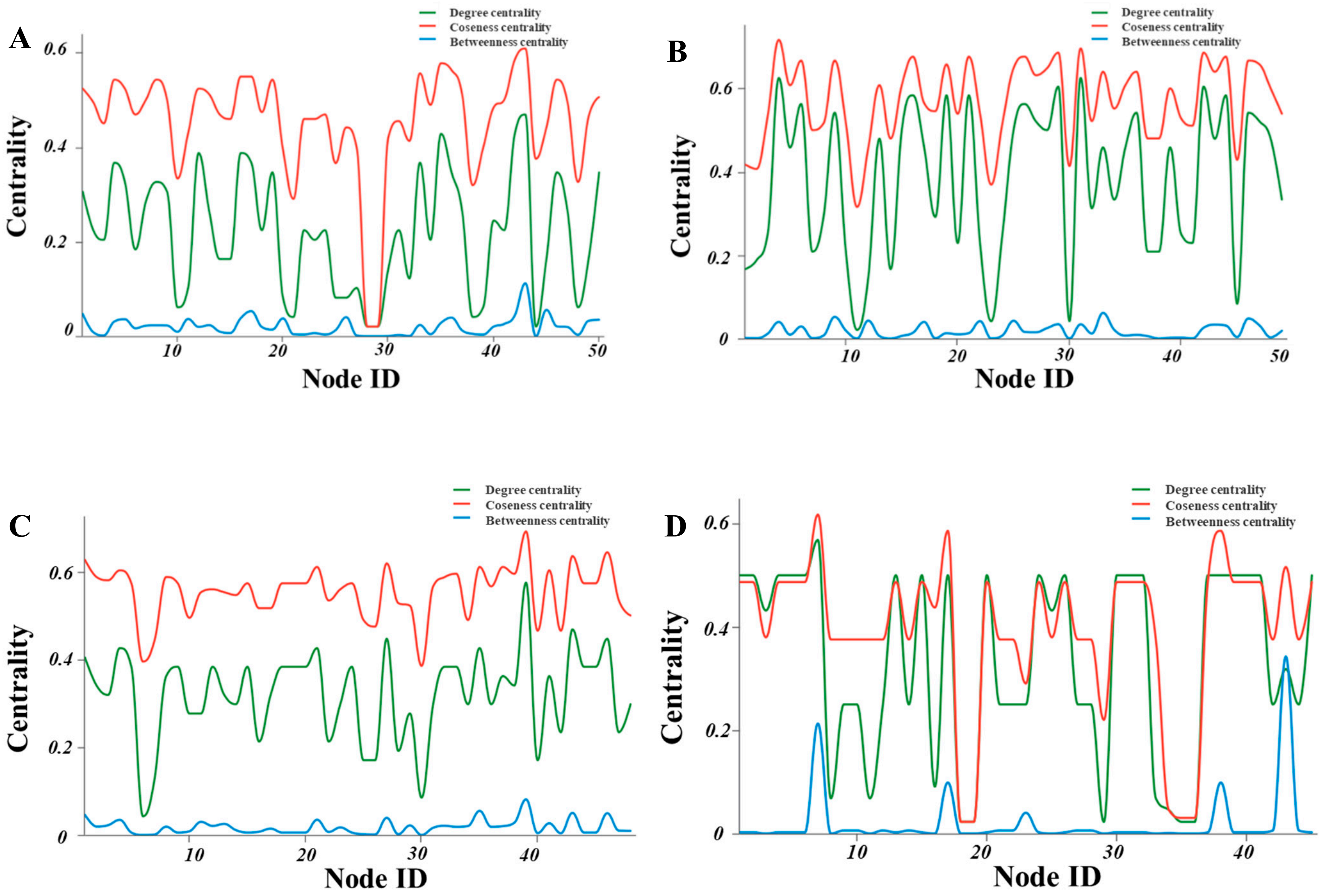

Supplementary Fig. S8 Co-occurrence analysis of *Sirex noctilio* larval gut (A, C) and frass (B, D) microbiota co-occurring bacteria (A-B) and fungi (C-D) at the genus level in different regions.

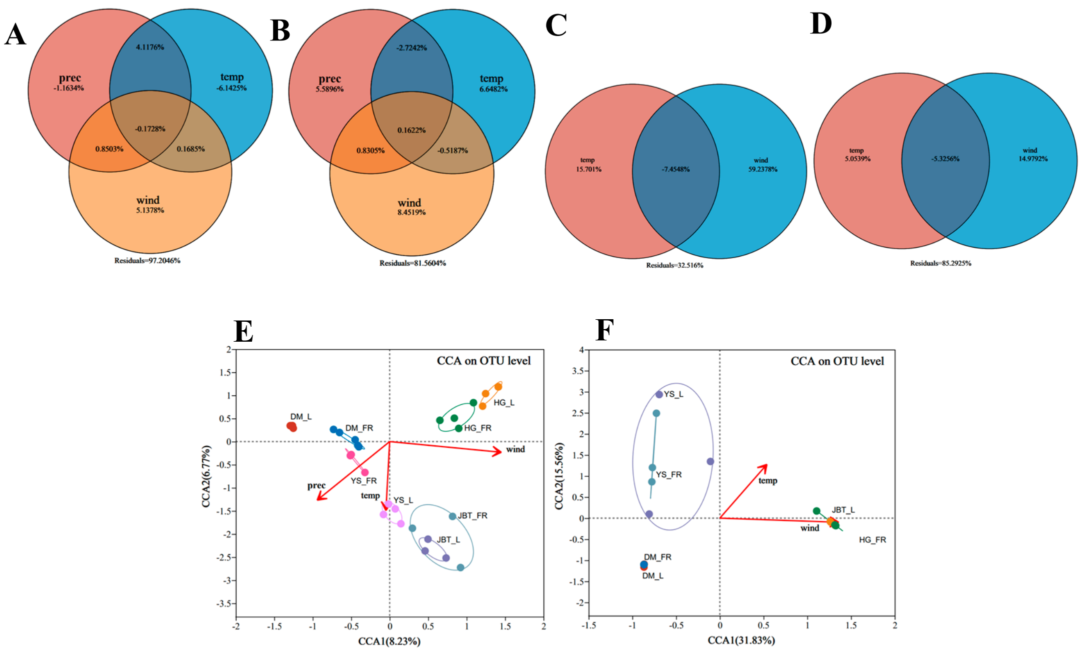

Supplementary Fig. S9 VPA analysis showing the relative interpretation rate of climatic factors on the variation of *Sirex noctilio* bacterial (A, B) and fungal (C, D) communities of frass (A, C) and larvae & frass (B, D) respectively. CCA analysis showing the effect of climatic factors on the distribution of *Sirex noctilio* bacterial (E) and fungal (F) communities of larvae and frass communities.

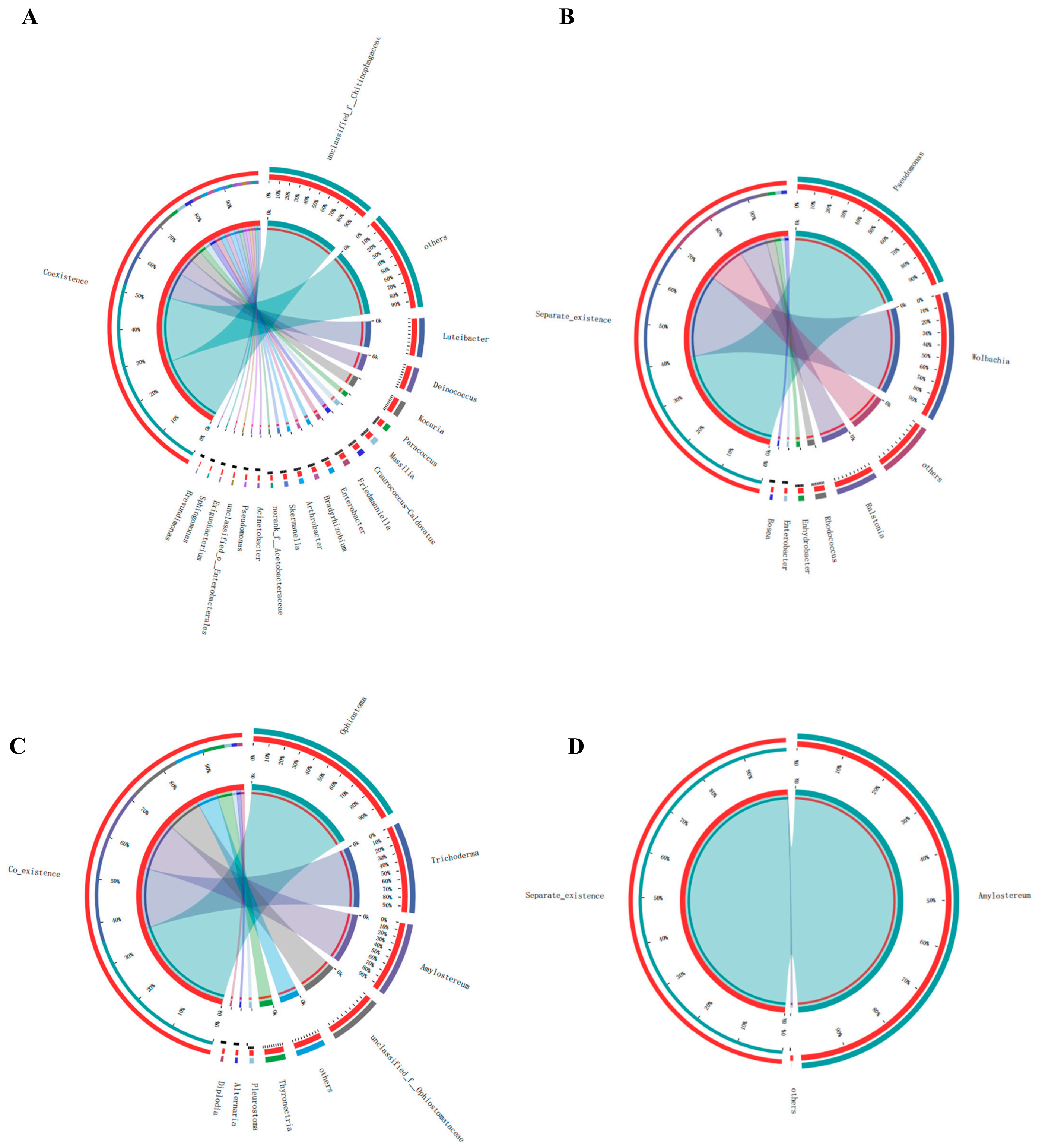

Supplementary Fig. S10 The circle graphs reflect the proportion of distribution of dominant genus in co-existence (A, C) and separate existence (B, D) sites samples. A-B, bacterial communities; C-D, fungal (F) communities.

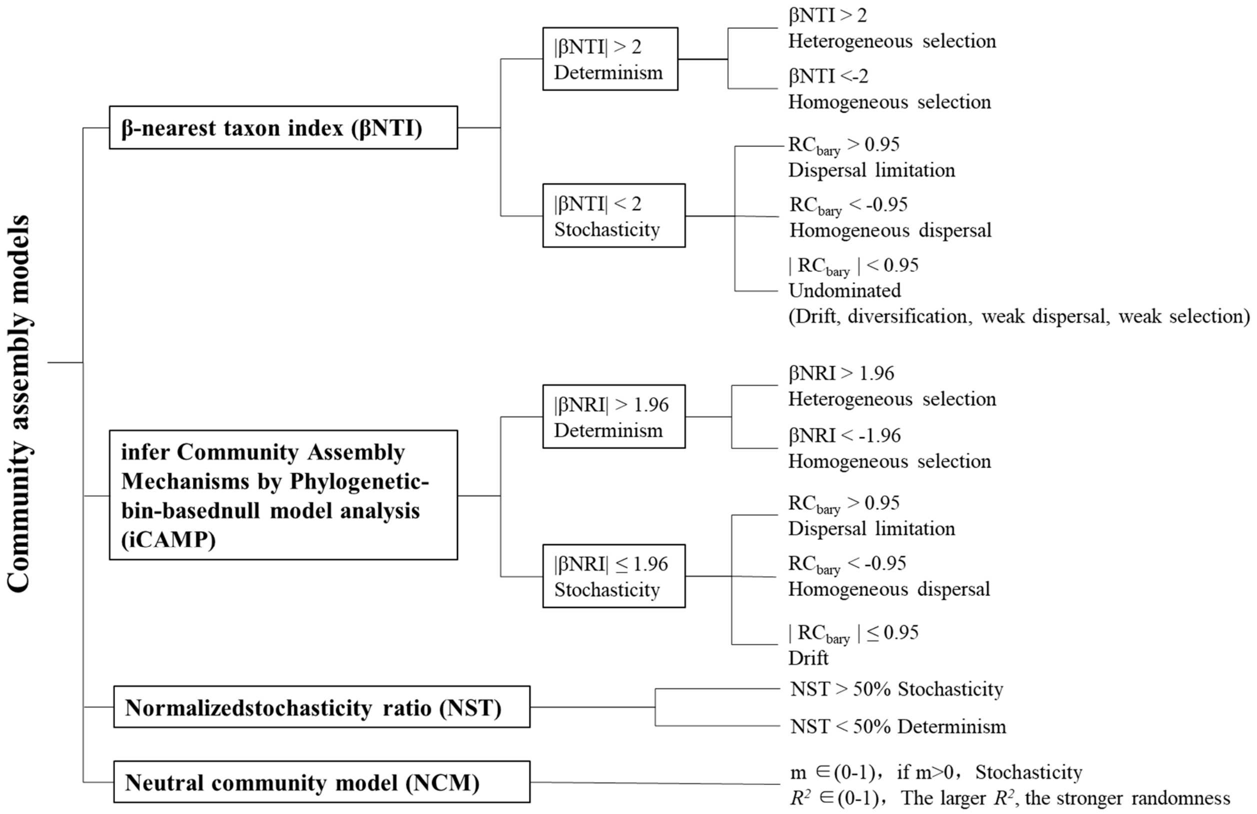

Supplementary Fig. S11 The framework of ecological models for assessing community assembly (modified from Stegen et al., 2015; Li et al., 2023)

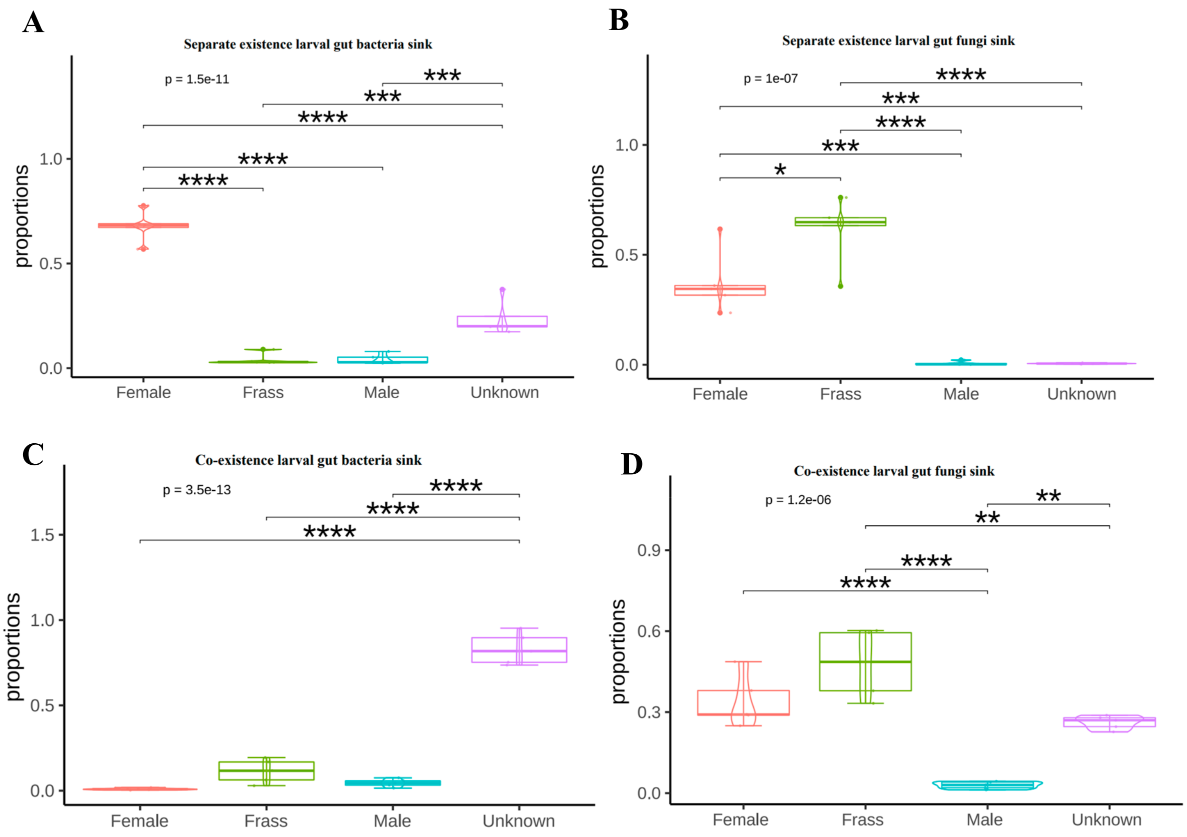

Supplementary Fig. S12 Source-tracking analysis graph reflect the proportion of different sources in a larval gut microbiota. A, C: bacterial communities; B, D: fungal (F) communities. ‘*’ was used to indicate p<0.05, ‘**’ was used to indicate p<0.01, and ‘***’ or ‘****’was used to indicate p<0.001.

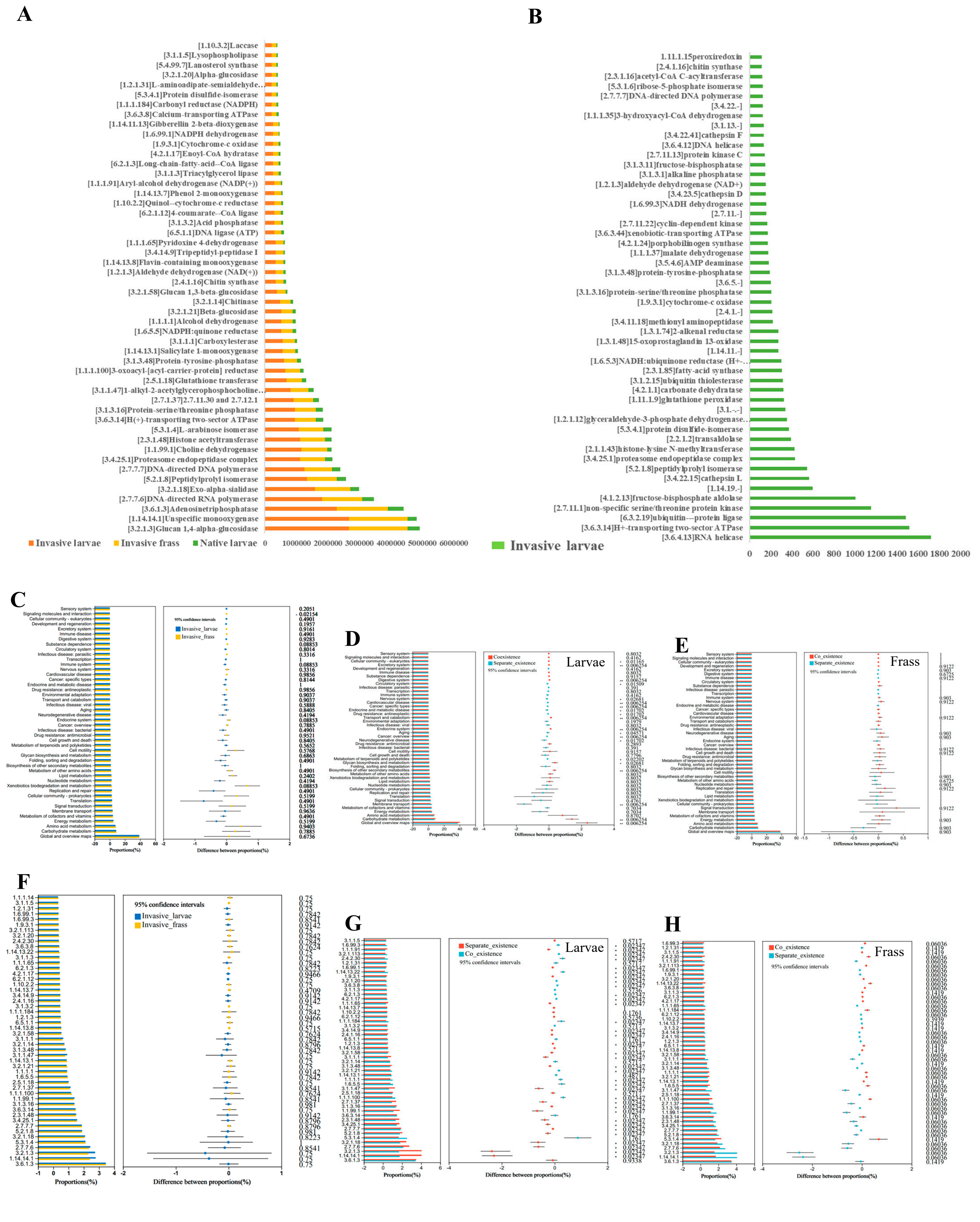

Supplementary Fig. S13 Functional prediction of amplicon (A, C, D, E, F, G, H) and metatranscriptome (B) sequences. The average number of predicted gene family counts.
